# Machine Learning-Driven Multi-Omics Analysis Identifies CHP2 as a Key PANoptosis-Related Dual-Function Biomarker in Colorectal Cancer

**DOI:** 10.3390/cells15050430

**Published:** 2026-02-28

**Authors:** Zetian Zhang, Xingyu Jiang, Xin Zhang, Fan Li

**Affiliations:** 1 The Key Laboratory of Zoonosis, Department of Pathogen Biology, Chinese Ministry of Education, College of Basic Medical Sciences, Jilin University, Changchun 130000, China; 2 The Key Laboratory for Bionics Engineering, Ministry of Education, Jilin University, Changchun 130000, China; 3 Engineering Research Center for Medical Biomaterials of Jilin Province, Jilin University, Changchun 130000, China; 4 Key Laboratory for Health Biomedical Materials of Jilin Province, Jilin University, Changchun 130000, China; 5 State Key Laboratory of Pathogenesis, Prevention and Treatment of High Incidence Diseases in Central Asia, Urumqi 830000, China

**Keywords:** calcineurin B homologous protein 2, PANoptosis, colorectal cancer, tumor immune microenvironment, machine learning-driven multiomics analysis, dual-function biomarker

## Abstract

**Highlights:**

**What are the main findings?**

- CHP2 was identified as a key tumor suppressor in colorectal cancer that inhibits cell proliferation and invasion by triggering PANoptosis (necroptosis, pyroptosis, and apoptosis).
- Low CHP2 expression characterizes a high-risk patient group associated with an immunosuppressive “cold” tumor microenvironment and poor clinical prognosis.

**What are the implications of the main findings?**

- CHP2 serves as a dual-function biomarker, providing a molecular basis for both prognostic risk stratification and the prediction of therapeutic response.
- CHP2 deficiency identifies patients resistant to standard chemotherapy who may selectively benefit from targeted inhibitors such as Ribociclib or Lapatinib.

**Abstract:**

The heterogeneity of colorectal cancer (CRC) represents a great challenge in therapy. We integrated multiomics and machine learning, interpreted by SHAP models to provide a clinical rationale, to identify Calcineurin B Homologous Protein 2 (CHP2) as a core candidate, which was further validated via in vitro and zebrafish models. The expression of CHP2 are decreased in CRC, which is associated with a poor prognosis and an immune suppressed “cold” TIME. Functionally, CHP2 overexpression inhibits cell growth and invasion by inducing PANoptosis. Clinically, specific CHP2 expression profiles discriminate patients at high risk that are resistant to standard chemotherapy (e.g., 5-FU) but sensitive to targeted inhibitors. CHP2 is a powerful dual-function biomarker—prognostic for survival and predictive for the response to therapy—that could lead to a personalized approach in treating drug-resistant CRC.

## 1. Introduction

Colorectal cancer (CRC) is a severe hazard to public health. It is the third most common type of cancer and the second most common cause of cancer-related death worldwide. There has been a significant increase in the number of cases in recent decades [1]. It causes approximately 10% of all malignancies and cancer-related deaths that are diagnosed each year worldwide. Despite the fact that CRC is the third most prevalent cancer in males and ranks second most frequently in females, women have a 25% lower incidence and fatality rate than men do [2]. These rates also exhibit geographical variations, with the highest figures reported in more developed nations [3]. Although early screening has decreased the incidence and mortality of CRC, 25–50% of individuals who receive an early diagnosis will later develop metastases, and 25% of patients present with advanced disease upon diagnosis [4]. The patients’ five-year survival rate who are in advanced CRC is barely 40%, even though first-line treatment options, including chemotherapy and targeted therapy, have improved. For those with metastatic colorectal cancer (mCRC), this risk is significantly lower, at only 15.6% [5]. CRC can be divided into four consensus molecular subtypes (CMSs) on the basis of genetic traits: CMS1 (MSI immune, 14%), CMS2 (canonical, 37%), CMS3 (metabolic, 13%), and CMS4 (mesenchymal, 23%) [6]. These subtypes vary in their prognosis and immune microenvironment [7,8]. Furthermore, CRC can be classified as either “cold” (pMMR/MSS) or “hot” (dMMR/MSI-H) tumors according to the features of the immune microenvironment. Mismatch repair deficiency/microsatellite instability-high (dMMR/MSI-H) cancers are categorized as “hot” tumors among CRC subtypes [9,10]. They are responsible for approximately 5% of mCRC cases and 15% of nonmetastatic CRC cases. The sensitivity of this subtype to immune checkpoint inhibitors (ICIs) is very high [11]. For example, the KEYNOTE-177 study (NCT025663002) [12] reported objective response rates (ORRs) ranging from 43.8% to 45.1%, establishing ICIs as the standard therapy for treating dMMR/MSI-H CRC. More recently, a study by Thierry Andre et al. [13] reported the phase III CheckMate 8HW trial (NCT04008030), which led to significant improvements with a dual immunotherapy regimen, integrating nivolumab and ipilimumab, for the first-line treatment of dMMR/MSI-H mCRC. The combination therapy resulted in substantial enhancements in both progression-free survival (PFS) and the overall response rate (ORR). However, the aforementioned treatment advancements have focused primarily on the dMMR/MSI-H CRC subtype, which is characterized by a “hot” immune microenvironment and significant immunogenicity. The more common “cold” tumor subtype, pMMR/MSS CRC, which is resistant to ICIs, nonetheless has poor treatment efficacy. Several novel therapeutic approaches have been investigated recently. One example is the use of a PD-1/VEGF bispecific antibody in conjunction with the FOLFOXIRI regimen for the first-line treatment of pMMR/MSS mCRC [14]. Another approach involves the use of the SIRT2 inhibitor AGK2 with an anti-PD-1 antibody. This combination aims to change the “cold” tumor microenvironment into a “hot” one, a strategy shown to significantly lower the tumor burden and increase survival [10]. Repurposing aclidinium bromide, a medication initially prescribed for chronic obstructive pulmonary disease, to target CERS3 activity in colon cancer is another innovative strategy [15]. Nevertheless, all of the aforementioned treatment strategies are currently in clinical or preclinical trial stages and are associated with varying degrees of side effects and drug resistance. Thus, identifying genes in CRC cells that can induce different types of cell death and monitor drug resistance is important for the treatment of CRC. Such a result is critical to allow early identification of therapy resistance and drive the development of new CRC therapies.

Regulated cell death (RCD) is a crucial aspect of cancer treatment because it removes cancerous cells and affects the treatment response [16]. PANoptosis is a new type of RCD. It is characterized by the simultaneous activation of pyroptosis-, necroptosis-, and apoptosis-related pathways [17]. PANoptosis is orchestrated by the multiprotein complex PANoptosome [18] and was originally described in 2019 [19]. The molecular details of PANoptosis are currently being elucidated. Four major PANoptosome-forming complexes, which include sensors such as ZBP1, AIM2, RIPK1, and NLRP12, have been described thus far [20,21,22,23,24]. In addition, Karki et al. [25] reported that TNF-α- and IFN-γ-mediated PANoptosis induces the JAK/STAT1-IRF1-iNOS/NO signaling pathway. While the complex biology of PANoptosis remains challenging to fully elucidate, its functional relevance has been firmly established in several malignancies. For example, NFS1 phosphorylation, which triggers the PANoptosis pathway, was found to reduce the sensitivity of CRC to oxaliplatin [26]. In contrast, interferon regulatory factor 1 (IRF1) can inhibit CRC by modulating PANoptosis [27]. The p53-regulated gene, which is a UNC5 family member, UNC5D, has been found to induce DNA or RNA damage through this pathway, which in turn could contribute to chemosensitivity in tumors [28]. The expression trends of PANoptosis-related genes (PANRGs) as predictive biomarkers are gradually being recognized. Multiple studies have shown that PANRGs are correlated with patient survival rates and the tumor immune microenvironment (TIME). Furthermore, these patterns appear to predict responses to both chemotherapy and immunotherapy [29,30,31]. Moreover, several core PANoptosis-regulating genes, such as CDK1 [32], TIMP1, CDKN2A [29], RDX [33] and LPCAT1 [34], influence the TIME in various cancers. Recent data suggest that PANoptosis can remodel the TIME, improve immune cell infiltration, and possibly prevent treatment resistance [35]. However, it remains unclear whether PANoptosis can transform the immunologically “cold” phenotype of CRC into a “hot” phenotype. One of the primary objectives of this study is to answer this question.

The relationship between PANoptosis and CRC was the focus of our comprehensive bioinformatic evaluation in the present study. Calcineurin B homologous protein 2 (CHP2) is the key component for the diagnostic and prognostic model that we initially identified and screened from a set of PANRGs dysregulated in CRC via a number of machine learning algorithms. CHP2 is a key member of the calcium-binding protein subfamily whose critical biological function is to act as a regulator of Na+/H+ exchanger 1 (NHE1), thereby playing an important role in maintaining intracellular pH homeostasis [36]. Interestingly, CHP2 is differentially expressed in some cancers in opposite manners. It is known to be a highly expressed oncogene in various cancers, including non-small-cell lung carcinoma [37], ovarian carcinoma [38], breast carcinoma [39] and prostate carcinoma [40]. However, there is not much information about its role in CRC because the limited data that are available suggest that it might be downregulated [41]. Two findings were confirmed by our comprehensive analysis. First, CHP2 is downregulated in CRC, which is highly correlated with an immunosuppressive “cold” tumor microenvironment and poor patient prognosis. Second, CHP2 overexpression inhibits CRC cell proliferation and invasion by activating multiple potential RCD pathways. However, CRC cells are resistant to broadly standard chemotherapies but remain sensitive to certain targeted agents. This was a pivotal finding in enabling us to realize the central clinical value of this risk model: a prediction of an intricate ‘‘selective resistance’’ phenotype. Low-CHP2 tumors illustrate this well. They are generally impervious to almost all conventional treatments, including “5-FU”. Paradoxically, this subset is extremely sensitive to a few specific targeted agents, uncovering potential “synthetic lethality” therapeutic options. These critical bioinformatic predictions were later confirmed via preliminary in vitro and in vivo experimental validation. In conclusion, this report identifies CHP2 as a major regulator of the immune contexture and drug response in CRC. This study develops a PANoptosis-based risk signature that provides a new and translatable approach for accurate molecular classification and individualized treatment.

## 2. Materials and Methods

### 2.1. Data Downloading and Processing

We combined a set of genes linked to pyroptosis [42], apoptosis [43], and necroptosis [44] for this research. These genes are now known as PANoptosis-Related Genes (PANRGs) (Table S1). Using the search term “colorectal cancer”, we then downloaded CRC datasets from the Gene Expression Omnibus (GEO, https://www.ncbi.nlm.nih.gov/geo/, accessed on 8 October 2025): GSE14333 (290 samples from CRC patients) [45], GSE17536 (samples from 177 tumor patients) [46], GSE20916 (105 tumor tissues and 40 normal tissues) [47], GSE39582 (566 tumor tissues and 19 normal tissues) [48], and GSE9348 (70 tumor tissues and 12 healthy tissues) [49]. Additionally, we downloaded data from The Cancer Genome Atlas (TCGA), including data from the TCGA-COAD (483 tumor tissues and 41 normal tissues) and TCGA-READ (167 tumor tissues and 10 normal tissues) cohorts. Ethical approval and informed consent are not needed because we obtained all the data from publicly accessible GEO and TCGA databases.

### 2.2. Differential and Functional Enrichment Analysis of PANRGs

The relationships between the aforementioned three gene sets were visualized via the “VennDiagram” R package. We conducted functional enrichment analysis on the merged database comprising all cohorts. This merged, batch-corrected matrix was used for descriptive transcriptome-wide analyses, such as differential expression and functional enrichment). Model training and evaluation were thus performed under a cross-validation scheme using out-of-fold predictions; each sample was evaluated on a model that was not trained on itself for avoiding information leakage in subsequent machine-learning (ML) evaluation. We subsequently identified differentially expressed PANRGs (DE-PANRGs) between CRC and adjacent normal tissues. Functional enrichment analysis was conducted on these DE-PANRGs via the R packages “clusterProfiler”, “org.Hs.eg.db”, “enrichplot”, and “ggplot2”. Significant enrichment was defined as GO and KEGG enrichment with an adjusted *p* value < 0.05.

### 2.3. Integrated Machine Learning Approach for DE-PANRGS Selection

To identify the most robust diagnostic markers from the set of DE-PANRGs, three different machine learning algorithms were applied: support vector machine recursive feature elimination (SVM-RFE), least absolute shrinkage and selection operator (LASSO) and random forest (RF). **SVM-RFE**: The SVM-RFE algorithm recursively builds models and eliminates features with the lowest contribution to find the optimal group of predictive genes. This analysis was conducted with the R packages “e1071” and “caret” to classify CRC patients and healthy controls. **LASSO:** The model was developed via 10-fold cross validation via the “glmnet” package to select a panel of feature genes for CRC diagnosis. **Random forest (RF):** RF is constructed via the “randomForest” package. Feature selection methods were performed in the cross-validation procedure in order to minimize optimistic bias. To improve robustness, the final key genes were obtained according to the consensus of the three algorithms. The importance of each DE-PANRG was ranked on the basis of the Gini impurity index to screen for feature genes. We selected the genes identified by these machine learning algorithms as the key DE-PANRGs for subsequent diagnostic analysis.

### 2.4. Comprehensive Evaluation of the DE-PANRGs

The chromosomal locations of the DE-PANRGs were then retrieved from the CNV-disease database (CNVD; http://bioinfo.hrbmu.edu.cn/CNVD/, accessed on 8 October 2025) and visualized via the R package “circlize”. To assess their diagnostic potential, we generated receiver operating characteristic (ROC) curves and areas under the curve (AUCs) via the R package “pROC”.

### 2.5. Immune Landscape of the Key Genes

CIBERSORTx (https://cibersortx.stanford.edu/, accessed on 8 October 2025) is a type of deconvolution algorithm. The relative proportions of twenty-two subtypes of infiltrating immune cells in the TIME of CRC were inferred (Table S2). Spearman’s rank correlation analysis was used to assess the relationships between key gene expression and immune cell abundances, with the results visualized via “ggplot2”. Correlations of gene expression with a curated set of immune-related genes were evaluated (Table S3).

### 2.6. GSVA and ceRNA Network Construction for the Key Genes

To investigate the key gene-associated biological pathways, GSVA was performed via the “GSVA” and “GSEABase” R packages, referencing the “c2.cp.kegg.v2023.1.Hs.symbols.gmt” gene set from MSigDB (https://www.gsea-msigdb.org/gsea/msigdb, accessed on 8 October 2025). Pathways with a modified *p* value of less than 0.05 were considered significant. To further elucidate potential regulatory mechanisms, a competing endogenous RNA (ceRNA) network comprising protein-coding mRNAs and noncoding RNAs crucial for tumorigenesis was constructed. Candidate miRNAs that target key genes were identified via cross-referencing with miRanda (http://www.microrna.org/, accessed on 8 October 2025), miRDB (http://www.mirdb.org/, accessed on 8 October 2025), and TargetScan (http://www.targetscan.org/, accessed on 8 October 2025). Long noncoding RNAs (lncRNAs) predicted to sponge these miRNAs were identified via SpongeScan (http://mirtoolsgallery.tech/mirtoolsgallery/node/1798, accessed on 8 October 2025). The resulting mRNA-miRNA-lncRNA ceRNA network was then integrated and visualized via Cytoscape software (v3.10.4).

### 2.7. Machine Learning Model Evaluation and SHAP Analysis

To examine the capacity of key genes to between tumors and normal samples, we employed a series of machine learning algorithms, such as KNN, RF, PLS, DTs, SVM, logistic regression, GBM, neural networks, and XGBoost. The model performance was evaluated by cross-validation and the AUC values were computed based on out-of-fold predictions in order to reduce a potential overfitting in the evaluation, and to not evaluate samples by models trained with those samples. The models were compared using ROC curves and their AUC, the best cross-validated AUC model was taken forward for downstream interpretation. To improve interpretability, we applied SHapley additive explanations (SHAP) on the final selected model and created SHAP summary, dependence and decision plots. SHAP delivers a post hoc model-specific explanation (feature attribution) by measuring the contribution of each input gene to the predicted probability at the global (cohort-level) and local (individual-prediction) interpretation levels. SHAP summary (bar plot) shows global feature importance sorted by SHAP value. And we analyzed pairs of genes with SHAP dependence plots (gene expression is x-axis and SHAP is y-axis, the gene expression interacting with the given gene is highlighted by color). We additionally follow the prediction path for a single prediction with SHAP decision plots. This combined analysis identified CHP2 as the top gene. All the analysis were conducted in R using the packages “Caret”, “DALEX”, “ggplot2”, “kernlab”, “kernelshap”, “klaR”, “pROC”, “randomForest”, “shapviz”, and “xgboost”.

### 2.8. Comprehensive Analysis of CHP2

Herein, we systematically investigated the expression, clinical relevance, and therapeutic implication of CHP2 in CRC. Firstly, we confirmed the difference in expression between tumor and normal samples in TCGA (COAD + READ) dataset. Secondly, we studied protein expression and its correlation with the clinical stages of the CRC patients in the UALCAN database [50]. Next, we investigated the prognostic value of CHP2 and its association with OS in CRC patients by the K-M plotter database, to assess its correlation with OS. To identify biological functions, we performed GSEA via the KEGG pathway database. Additionally, drug sensitivity analysis was executed via the R package “oncoPredict”. The training dataset (198 chemotherapeutic drugs) was retrieved from GDSC2. We computed the IC50 for each sample via the calcPhenotype function, where *p* < 0.05 was considered significant.

### 2.9. Cell Culture

Two CRC cell lines, HCT116 (BDBIO, Shanghai, China, C5229) and SW480 (BDBIO, Shanghai, China, C5233), and a normal human colorectal cell line, NCM460 (BDBIO, C5710), were cultivated in DMEM (Sigma-Aldrich, Shanghai, China D6429) supplemented with 1% penicillin-streptomycin-amphotericin B (Servicebio, Wuhan, China, G4015) and 10% FBS (Gibco, Shanghai, China, 10270106) at 5% CO_2_ and 37 °C. At 80–90% confluence, we detached the cells with 0.25% trypsin-EDTA (MCE, Shanghai, China, HY-K3007), neutralized them, centrifuged them at 300× *g* for 5 min, and subcultured them at a 1:6 ratio every 3–4 days for HCT116 and NCM460 cells and at a 1:4 ratio for SW480 cells. We selected these lines to cover the major genomic landscapes of CRC, as HCT116 harbors microsatellite instability (MSI) with wild-type p53, while SW480 is microsatellite stable (MSS) with mutant p53.

### 2.10. Lentivirus Infection

The CHP2-overexpression plasmid, plv-EF1a-CHP2-3*Flag-CMV-luc-P2A-Puro, and negative control vector were designed and manufactured by Shanghai Bio-Lifespan Co., Ltd., Shanghai, China. The plasmids were packaged in lentiviruses according to standard protocols. These lentivirus-packaged plasmids were named LV-CHP2-OV and LV-NC. HCT116 and SW480 cells were seeded in 6-well plates and incubated for 24 h until they reached 30–40% confluence. Then, we incubated the cells with LV-CHP2-OV or LV-NC at 1.25 × 10^6^ TU/mL with 6 μg/mL polybrene (Beyotime, Shanghai, China, C0351) for 24 h. To generate stable cell lines, we added 2 μg/mL puromycin (Beyotime, Shanghai, China, ST551) for one week. To validate the cell transfection experiment, we perfumed three independent Western blot experiment (Figure S1). The sequence of the CHP2 gene and the structure of the plasmid are provided in Table S4 and Figure S2.

### 2.11. siRNA Transfection and Knockdown Efficiency Validation

For rescue experiments, three candidate small interfering RNAs (siRNAs, 1, 2 and 3) against the CHP2 sequence were synthesized by Shanghai Bio-Lifespan Co., Ltd. The stable CHP2 overexpression cell lines were plated in six-well plates and transfected with these siRNAs using Lipo 8000 (Beyotime, C0533) based on the manufacturer’s protocol. All knockdown validation assay were independently conducted for three times to ascertain the reproducibility. Efficiency of silencing was determined by Western blot at 48 h post-transfection. Among these, siRNA-3 showed the strongest and most consistent inhibition of CHP2 protein expression, and it was used in subsequent functional rescue assay. The specific sequences of the siRNAs are 5’-CACTGGACAGGAATAAGAA-3’, 5’-GAAGGAACAAACTTCACTA-3’, and 5’-GGAGTTCACCAAGTCCTTA-3’ [37]. And the results of the knockdown experiments are presented in Figure S3.

### 2.12. RNA Extraction and qRT-PCR

Total RNA was obtained from NCM460, HCT116, and SW480 cells with a Total Cellular RNA Extraction Kit (Tiangen, Beijing, China, DP419). For mRNA and lncRNA, cDNA was generated via aBeyoRT™ II First Strand cDNA Synthesis Kit with gDNA Eraser (Beyotime, Shanghai, China, D7170S). For miRNA, we used the stem-loop method to generate cDNA according to the instructions of the miRNA 1st Strand cDNA Synthesis Kit (by stem-loop) (Vazyme, Nanjing, China, MR101-01). qRT-PCR was performed on an ABI 7300 Plus Real-Time PCR System (Applied Biosystems, Waltham, MA, USA) with ChamQ Universal SYBR qPCR Master Mix (Vazyme, Nanjing, China Q711). The expression of mRNA and lncRNA was normalized to β-actin and the miRNA expression was normalized to U6. Gene expression was measured via the 2^−ΔΔCt^ method on the basis of four biological replicates to ensure statistical reliability. The primers were shown in Table S5.

### 2.13. Cell Proliferation

We utilized a CCK8 assay (Beyotime, Shanghai, China, C0037) to determine cell viability, with three replicates. The impact of CHP2 overexpression on cell proliferation was assessed via Click-iT™ Plus OPP Alexa Fluor™ 488 (Thermo Fisher, Waltham, MA, USA, C10456). 1 × 10^5^ HCT116, HCT116-CHP2-OV, SW480, and SW480-CHP2-OV cells were inoculated into each well of 24-well plates and treated with 50 μM EdU for 2 h. After washing with PBS, the cells were fixed with 4% paraformaldehyde for 30 min and treated with 2 mg/mL glycine for 5 min. After washing again with PBS, the cells were permeabilized for 10 min, followed by another PBS wash. For EdU detection, the cells were stained with 100 μL of 1 × Apollo^®^ reaction mixture for 30 min in the dark and then washed sequentially with permeabilization buffer (3 × 10 min), methanol (2 × 5 min), and PBS (1 × 5 min). Nuclei were counterstained with 1× Hoechst 33342 reaction mixture (1:2000 dilution) for 30 min in the dark, followed by three washes with PBS (500 μL each). Images were taken via a fluorescence microscope (Olympus IX71, Tokyo, Japan), and the ratio of EdU-positive cells was determined via ImageJ (NIH, Bethesda, MD, USA) by counting Apollo-positive (green) and total (blue) nuclei. Each EdU assay was repeated three times independently (Figure S4).

### 2.14. Drug Sensitivity Assay

To assess the ability of CHP2 to predict treatment response, we conducted drug sensitivity assays using HCT116 and SW480 cell lines. Cells were seeded in 96-well plates at a density of 5 × 103 cells/well and cultured for 24 h for attachment. The cells were treated with series concentrations of traditional chemotherapeutic drugs (5-fluorouracil, Oxaliplatin, and Irinotecan) and targeted agents (Ribociclib and Lapatinib) for 48 h. All of the drugs are purchased from MCE, Shanghai, China. Cell viability was measured using the CCK-8 assay (Beyotime, C0037). To confirm reproducibility, all of the experiments were carried out 3 times, and the dose–response curves obtained were used for the analysis of the drug sensitivity profiles between Control (low-CHP2) and CHP2 overexpression.

### 2.15. Western Blotting

Proteins were extracted via the Column Tissue & Cell Protein Extraction Kit (Epizyme, PC201). After the protein concentration was determined with a BCA protein assay kit (Beyotime, P0010), identical amounts of proteins were subjected to SDS-PAGE and transferred onto PVDF membranes, which were subsequently incubated with primary antibodies against CHP2 (Boster, Wuhan, China, A08478-2, 1:1000), p-MLKL (MCE, HY86069, 1:1000), GSDMD (N-terminal) (MCE, HY-P85810, 1:1000), Cleaved Caspase-3 (HY-P86370) and β-actin (Servicebio, ZB15001-HRP, 1:2000). We washed membranes by TBST and then incubated them an secondary antibody solution for 24 h at 4 °C. The signals were detected via ECL reagents (Bioswamp, Wuhan, China, PW9209) and quantified with ImageJ. Each blot image represents three independent replicates. Uncropped blots are provided in the Blot Transparency section of the Supplementary Information (Figures S5 and S6).

### 2.16. Immunofluorescence

The cells grown on the slides were fixed with 4% paraformaldehyde for 30 min and permeabilized with 0.5% Triton X-100 (Beyotime, P0096) for 15 min. Following blocking with QuickBlock™ Blocking Buffer (Beyotime, P0260) for 30 min, the cells were incubated with an anti-CHP2 antibody (1:200) overnight at 4 °C and with an AF488-conjugated goat anti-rabbit IgG (H + L) secondary antibody (Beyotime, P0176) for 1 h. After nuclear counterstaining with DAPI (Beyotime, C1002) for 10 min, the slides were mounted and observed via a fluorescence microscope (Olympus IX71, Tokyo, Japan) for CHP2 expression. Data were representative of 3 independent experiments (Figure S7).

### 2.17. Transwell Migration and Invasion Assays

We used transwell chambers (12.0 μm, PC membranes, Miraclebio, Beijing, China MRC-C-23011) to assess cell migration and invasion. HCT116, HCT116-CHP2-OV, SW480, and SW480-CHP2-OV cells were resuspended in serum-free medium. Matrigel (150 μg/mL, prediluted in chilled serum-free medium) was applied to the inserts for the invasion assay and incubated at 37 °C for 4–5 h. In the migration assay, uncoated inserts were used. The cells (1 × 10^5^ in 200 μL of serum-free medium) were loaded into the upper chamber, and 600 μL of 10% FBS medium was added to the lower chamber. After 24 h, nonmigrated cells were removed, and migrated cells were incubated with 4% paraformaldehyde (Bioharp, Hefei, China, BL539A) for 15 min and stained with 0.1% crystal violet (Servicebio, G1014). Five random fields per insert were imaged, and the cells were counted with ImageJ (Figure S8).

### 2.18. Flow Cytometry

After transfection with OV-CHP2 or OV-NC, CRC cells were harvested via trypsinization and subjected to Annexin V-APC/7-AAD (Elabscience, Wuhan, China E-CK-A218) double staining. Dead cells were analyzed via flow cytometry (BD Bioscience, US). We split all cells into 5 groups: Single-stained and double-stained group, Control group, Lipo8000 group, O-NC group, and O-CHP2 group. Each group was performed three times (Figure S9).

### 2.19. In Vivo Functional Validation

SW480 cells and SW480-CHP2-OV cells were stained with CM-Dil (Bestbio, Nanjing, China, BB441924) according to the instructions. Subsequently, 48 hpf wild-type AB strain zebrafish (Qingdao Benyue Biotechnology Co., Ltd., Qingdao, China) were randomly selected for microinjection with an injection volume of approximately 5 nl to establish a cancer cell xenograft model and were divided into the control group and the CHP2-OV group. Twenty zebrafish were processed in each group. After being treated for 72 h in a constant-temperature light incubator at 28 °C, zebrafish samples from each experimental group were collected and observed under a fluorescence microscope, and data were collected. The fluorescence area and intensity in the zebrafish were measured via software. For the cell migration experiment, tumor cell migration was observed through a fluorescence microscope. Taking the zebrafish yolk sac injection point as the origin, a straight-line distance was drawn according to the surrounding transplanted tumor (the straight-line distance from the injection point to each labeled cell was the migration distance), and software was used to measure the migration distance of each migrating cell and the number of migrating cells.

### 2.20. Statistical Analysis

Data were collected from a minimum of three separate experiments from individual cells and are shown as the means ± standard deviations. To test differences between two groups, an unpaired two-tailed Student’s *t* test was used. One-way analysis of variance (ANOVA) and Tukey’s test were used for multiple group comparisons. ImageJ software was used to perform the fluorescence assays and Western blot experiments. Statistical analysis was performed via GraphPad Prism 10.0 (GraphPad Software, San Diego, CA, USA). *p* values < 0.05 were considered statistically significant in all analyses. * *p* < 0.05, ** *p* < 0.01, *** *p* < 0.001, **** *p* < 0.0001.

## 3. Results

### 3.1. Characterization of DE-PANRGs in CRC

**Figure 1:**
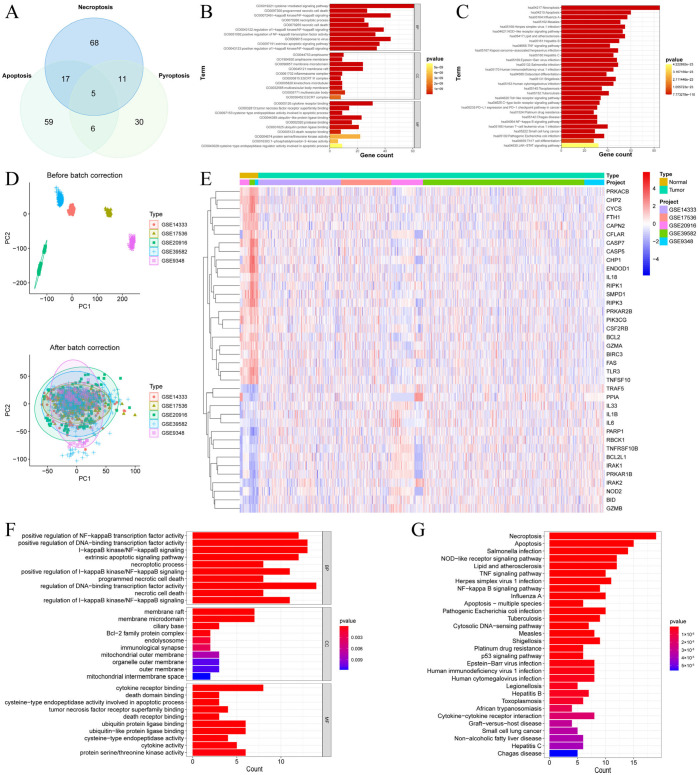
Characterization of DE-PANRGs in CRC. (**A**) Venn diagram illustrating the overlapping target genes for pyroptosis, apoptosis, and necroptosis, which collectively constitute PANoptosis. (**B**) GO terms of all PANRGs. (**C**) KEGG pathways of all PANRGs. (**D**) Boxplots depict the expression distribution across the integrated CRC datasets (GSE14333, GSE17536, GSE20916, GSE39582, and GSE9348) before and after normalization. (**E**) Heatmap of DE-PANRGs between CRC and normal tissues. (**F**) GO enrichment of DE-PANRGs. (**G**) KEGG pathway enrichment of DE-PANRGs.

We first mapped the gene targets in the three cell death pathways associated with PANoptosis (Figure 1A). Our analysis revealed 68 genes related to necroptosis, 59 related to apoptosis, and 30 related to pyroptosis. The fact that these groups share common gene targets points to a potential interplay and shared control mechanisms among the three cell death pathways. We subsequently performed a functional enrichment analysis for all these PANRGs. The GO analysis highlighted their major biological roles (Figure 1B). With respect to biological processes (BP), the PANRGs clustered heavily in the cytokine-mediated signaling pathway and the I-κB/NF-κB cascade, including its regulation (e.g., I-κB/NF-κB signaling regulation and positive NF-κB regulation). For CC, the genes were mainly localized to membrane microdomains and membrane rafts. In the Molecular Function (MF) category, enrichment was observed in cytokine receptor binding, death receptor binding, and ubiquitin-like protein ligase binding. KEGG pathway analysis (Figure 1C) further confirmed the involvement of these genes in necroptosis, apoptosis, and pyroptosis pathways, including the Toll-like and NOD-like receptor signaling pathways [51,52,53,54,55,56]. Notably, enrichment was also observed in cancer-relevant immune pathways (PD-1 checkpoint pathway and PD-L1 expression pathway) and TNF and NF-κB signaling pathways. Full GO and KEGG results are presented in Tables S6 and S7.

Next, five CRC GEO datasets were normalized (Figure 1D), and differential PANRG expression between CRC and normal sampled was analyzed. Utilized the thresholds of |log2 (fold change)| > 0.5 and adjusted *p* < 0.05, 38 DE-PANRGs were identified (Figure 1E, Table S8). Functional enrichment analysis of these DE-PANRGs (Figure 1F,G) revealed results consistent with those of the entire PANRG set. The enriched GO terms included NF-κB/I-κB pathway regulation, apoptosis, necroptosis (BP), membrane rafts and membrane microdomain structures (CP), death receptor binding, cytokine receptor binding, and ubiquitin/ubiquitin-like protein ligase binding (MF). Similarly, KEGG analysis confirmed enrichment in signaling pathways related to necroptosis, apoptosis, NOD-like receptor, NF-κB, TNF, and p53 (Figure 1G). The complete enrichment results are provided in Tables S9 and S10.

### 3.2. Identification of Six Key PANRGs for CRC Diagnosis

**Figure 2:**
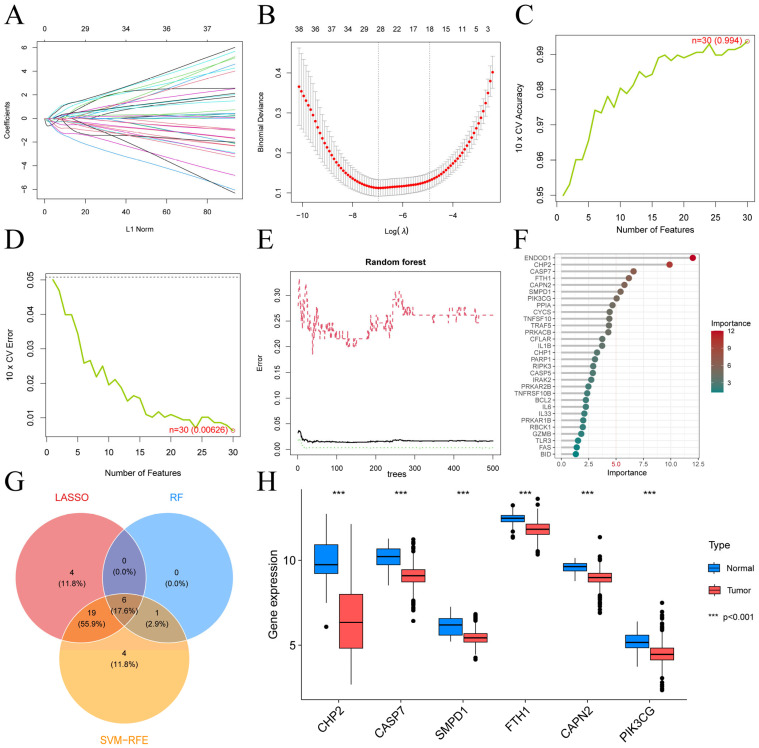
Identification of six important PANRGs in CRC. (**A**,**B**) LASSO logistic regression model based on 29 feature PANRGs. (**C**,**D**) SVM-RFE method, with the best subset including 30 key candidates (accuracy: 0.994; RMSE: 0.00626). (**E**,**F**) RF method, which pinpointed seven genes with an importance score > 5. (**G**) Venn diagram of PANRGs among the three algorithms. (**H**) Expression patterns of the six hub genes (CHP2, CASP7, SMPD1, FTH1, CAPN2, and PIK3CG) in CRC.

**Figure 3:**
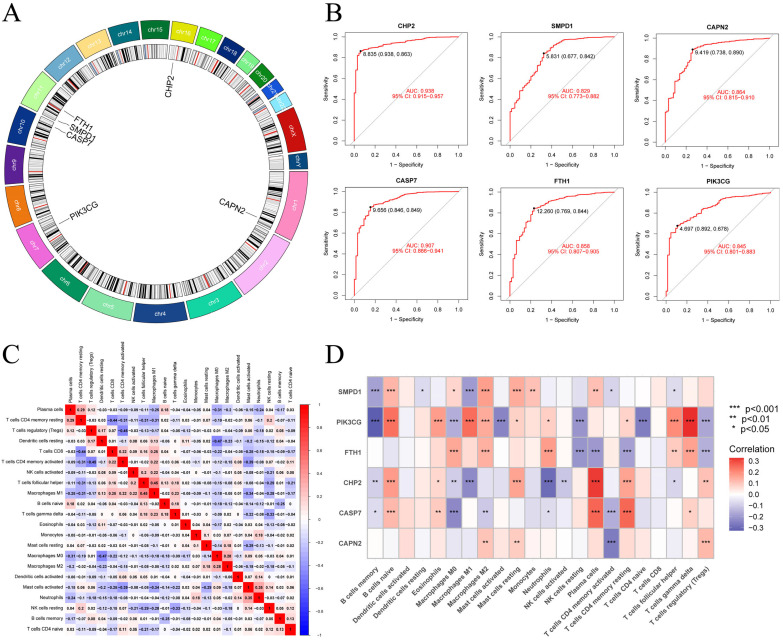
Chromosomal location, diagnostic performance, and immune landscape of the six key genes. (**A**) Circos plot of the chromosomal locations of the six key genes. (**B**) ROC curves used to evaluate the diagnostic performance of the six PANRGs. (**C**) Heatmap of the correlations among different tumor-infiltrating immune cells in CRC. (**D**) Heatmap displaying the correlations between the six PANRGs and infiltrating immune cells.

Given the significant molecular differences between CRC and normal tissues, we assessed the diagnostic potential of 38 DE-PANRGs via LASSO, SVM-RFE, and RF. The LASSO algorithm reduced the 38 DE-PANRGs to 29 candidate genes (Figure 2A,B; Table S11). The SVM-RFE algorithm revealed an optimal subset of 30 PANRGs with the highest classification accuracy of 0.994 and the lowest root mean square error (RMSE) of 0.00626 (Figure 2C,D; Table S11). We first depicted gene importance and flagged seven genes with scores > 5 via the RF algorithm **(**Figure 2E,F; Table S11). We then narrowed this list by intersecting it with two other algorithms. This step yielded six key diagnostic genes: CHP2, CASP7, SMPD1, FTH1, CAPN2, and PIK3CG (Figure 2G). All six genes were validated to be significantly differentially expressed between tumor and normal samples (Figure 2H). We performed ROC curve analysis and constructed ROC curves for their diagnostic value after locating them on chromosomes (Figure 3A). All six genes had AUCs > 0.8. CHP2 emerged as the top candidate with the best diagnostic performance (AUC = 0.938), suggesting that it is a promising biomarker for CRC identification (Figure 3B).

### 3.3. Immune Landscape Analysis of the Key PANRGs

To explore the potential role of the six identified key PANRGs in the TIME, we first applied the CIBERSORT algorithm to estimate the relative infiltration abundance of 22 types of immune cells in CRC samples. As an essential baseline, we characterized the inherent immune interactome in CRC by investigating the associations within these immune cell subsets (Figure 3C). This global immune map suggests a complex pattern of synergistic and antagonistic interactions. Specifically, the correlation analysis revealed that pro-inflammatory elements (M1 macrophages) had positive correlations with effector cells (CD8+ T cells). Whereas, significant negative correlations were found between immunosuppresive and effector populations, for instance, Tregs were significantly negatively correlated with activated memory CD4+ T cells and CD8+ T cells.

Based on this initial CRC immune scenery, we then examined the association of these six PANRGs with the immune microenvironment in CRC (Figure 3D). Among them, CHP2 demonstrated a prominent and intricate coorlation pattern with the estimated immune landscape, with its expression positively correlated with several adaptive immune and resting-state immune cells, particularly naive B cells, plasma cells and resting CD4 memory T cells (*p* < 0.001). Conversely, CHP2 expression was significantly negatively associations with multiple effector and pro-inflammatory immune cells, including M0 macrophages, neutrophils, and activated CD4 memory T cells (*p* < 0.001). Considering that CHP2 is downregulated in CRC, these findings suggest that reduced CHP2 expression is may linked to impaired adaptive immune activation and increased pro-inflammatory cell infiltration [57,58,59,60,61].

**Figure 4:**
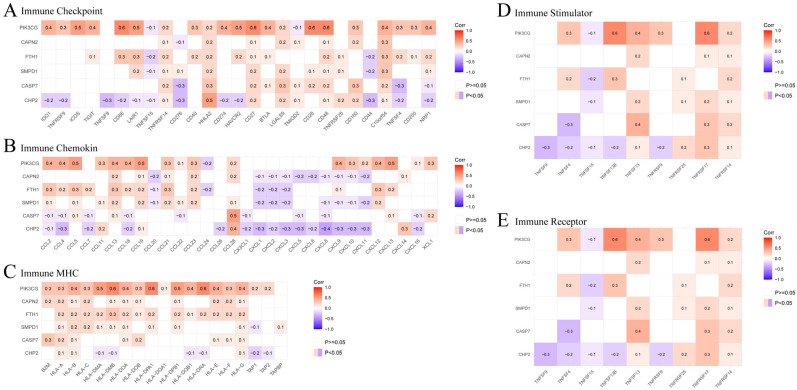
Correlation of core genes with key immune gene sets. Heatmaps of the correlations between the 6 core genes and key immune-modulatory genes: (**A**) immune checkpoint molecules, (**B**) chemokines, (**C**) major histocompatibility complex (MHC) molecules, (**D**) immune stimulators, and (**E**) immune receptors.

In order to investigate the immunoregulatory functions of key genes, we correlated their expression several immune gene sets: immune checkpoint, chemokine, MHC, and immune stimulators/receptors (Figure 4A–E). The results indicate that the expression of CHP2 was associated with a diverse and complex array of immune signaling molecules. For immune checkpoints (Figure 4A), CHP2 was positively correlated both with the immunoinhibitory molecule HHLA2 (B7-H7) and the immunostimulatory molecule CD40 (Corr = 0.5). In contrast, it was inversely associated with several major inhibitory and costimulatory molecules including CD276 (B7-H3), TNFSF9 (4-1BBL) and CD274 (PD-L1). This inhibitory phenotype was also mirrored in the immune chemokine system (Figure 4B), where CHP2 was most oppositely associated with the major proangiogenic and prometastatic molecule CXCL8 (IL-8) (Corr = −0.4). Conversely, CHP2 was positively correlated with the antitumor chemokines CCL28 (Corr = 0.4). With respect to MHC molecules (Figure 4C), the association with CHP2 is generally weak. However, it is noteworthy that it is negatively correlated with TAP1, a pivotal protein in the MHC class I antigen presentation pathway. The relationships to immune stimulators and their receptors (Figure 4D,E) further highlighted the complex association of CHP2 with immune-signaling pathways (negative for ligands including TNFSF9, TNFSF4; positive for receptors including TNFRSF17). Collectively, these correlative observations emphasize the intricate relationships between CHP2 and the multifaceted immune landscape and indicate that its expression level may correspond to certain immune signaling patterns in the CRC microenviroment.

### 3.4. Potential Biological Pathways and the Hypothetical ceRNA Regulatory Landscape

**Figure 5:**
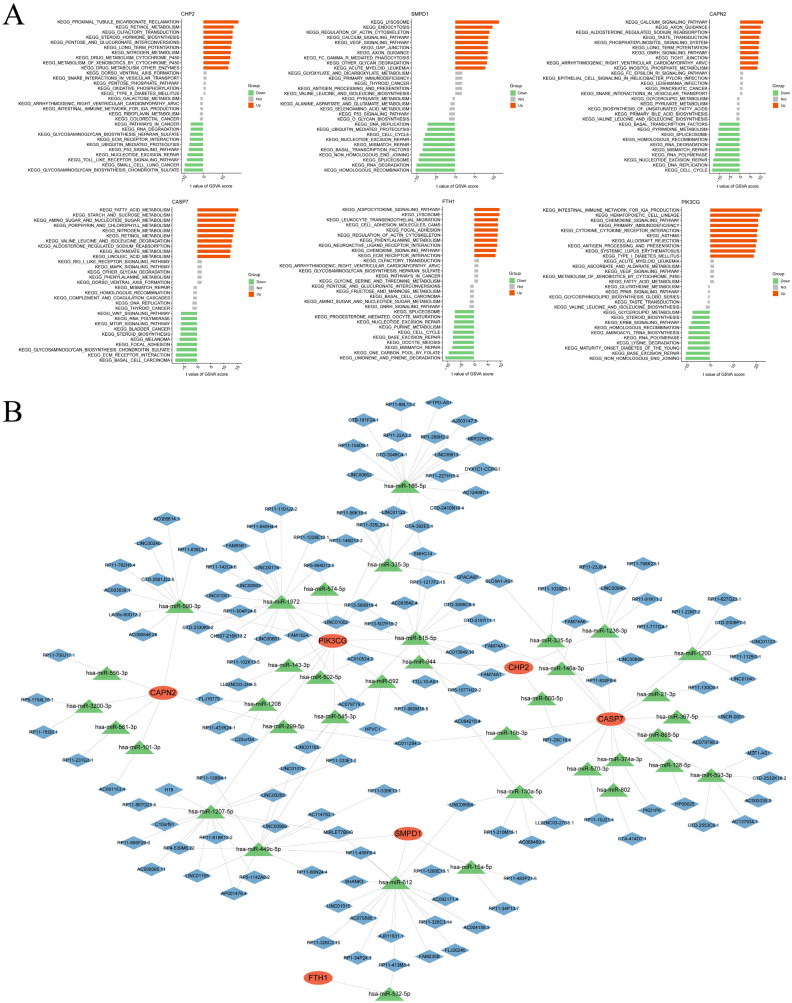
GSVA-KEGG pathway analysis and ceRNA network for the key genes. (**A**) GSVA-KEGG pathway analysis of the six core genes. (**B**) The constructed ceRNA network for the key genes.

At the pathway level, we applied GSVA the TCGA cohort and constructed an upstream ceRNA regulatory network to investigate the role of core genes (Figure 5). By GSVA (Figure 5A), the different CHP2 level was associated with different stages of pathway activation. Low CHP2 expression in samples was also clearly correlated with the progression of malignancy in tumors. Specifically, we observed significant activation (indicated by downregulated GSVA scores) of several pathways, including KEGG_PATHWAYS_IN_CANCER, KEGG_TOLL_LIKE_RECEPTOR_SIGNALING_PATHWAY, KEGG_UBIQUITIN_MEDIATED_PROTEOLYSIS, and KEGG_P53_SIGNALING_PATHWAY. Among the samples with high CHP2 expression, GSVA revealed two distinct functional modules. The first is related to controlling cell death, which is indicated by the activation of KEGG_NITROGEN_METABOLISM. We interpret this finding in the context of PANoptosis. This is because nitrogen metabolism feeds nitric oxide (NO) synthesis, and the iNOS/NO axis is a known trigger for PANoptosis [26]. Thus, high CHP2 expression may prime cells in a “ready-for-death” state. The second module was linked to metabolic reprogramming and drug resistance, as shown by activation of the KEGG_METABOLISM_OF_XENOBIOTICS_BY_CYTOCHROME_P450, KEGG_DRUG_METABOLISM_OTHER_ENZYMES, and KEGG_PROXIMAL_TUBULE_BICARBONATE_RECLAMATION pathways.

To explore the potential upstream regulation of the key genes, we established a ceRNA network comprising mRNAs, miRNAs, and lncRNAs (Figure 5B). MiRNAs targeting the key genes were identified by intersecting predictions from miRanda, miRDB, and TargetScan (Table S12), and lncRNAs predicted to sponge these miRNAs were retrieved from the SpongeScan database (Table S13). Focusing on CHP2, the network revealed interactions with several miRNAs, such as hsa-miR-146a-3p, hsa-miR-1236-3p and hsa-miR-660-5p. Among all candidate lncRNAs, FAM74A7 was the only one found to be directly lined with CHP2. Notably, LINC00689 also targets hsa-miR-1236-3p and hsa-miR-146a-5p, possibly forming a potential closed-loop regulatory structure involving CHP2. Then we performed qRT-PCR to validate the expression patterns of this predicted ceRNA network components in NCM460, HCT116 and SW480 cell lines. Our results revealed the inhibitory miRNAs hsa-miR-1236-3p, hsa-miR-146a-3p, and hsa-miR-660-5p were markedly upregulated and the lncRNAs LINC00689 and FAM74A7 were significantly downregulated in CRC cells (Figure S10). These bioinformatic inferences provide a hypothesis-generating framework for the multimodal regulation of CHP2. While we have performed preliminary qRT-PCR validation for these predicted candidates, more in-depth and extensive experiments are still required to better validate the potential interactions within this complex regulatory network.

### 3.5. Evaluating the Diagnostic Value of Key PANRGs via Machine Learning Models and SHAP Analysis

**Figure 6:**
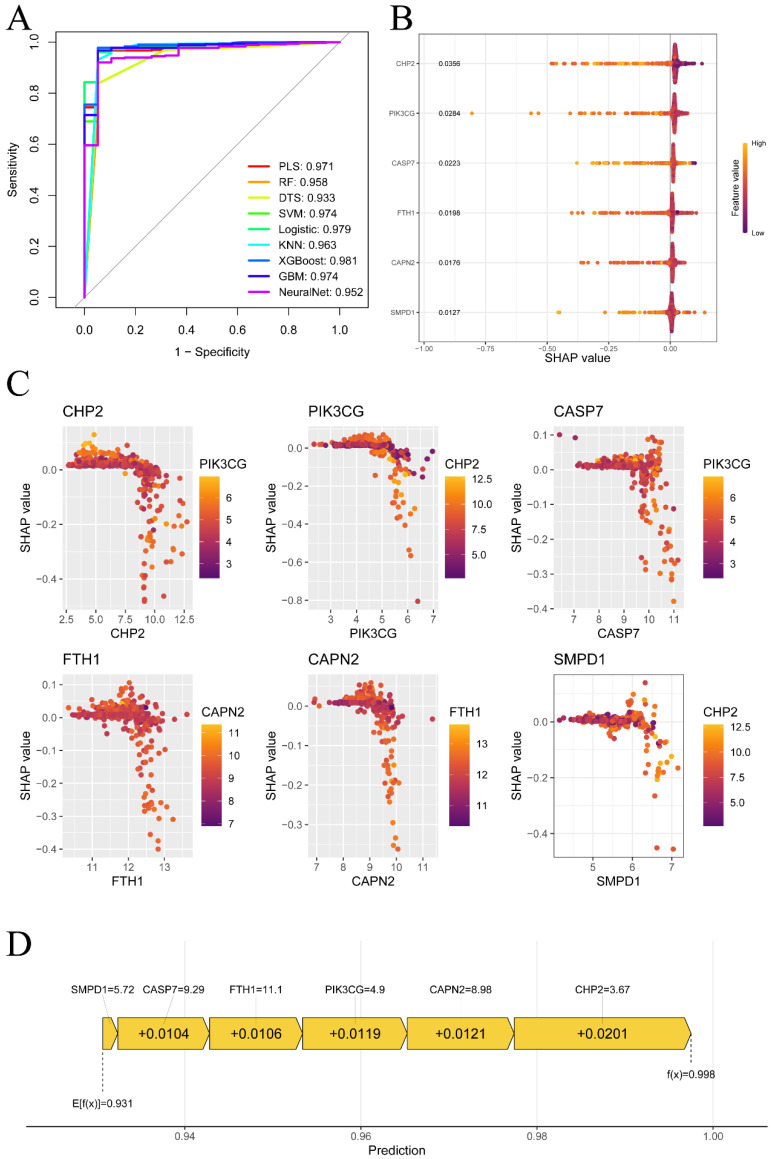
Diagnostic model evaluation of the key PANRGs via machine learning and SHAP analysis. (**A**) ROC curves for nine machine learning models evaluating the diagnostic value of the six core PANRGs, with AUC values provided in the legend. (**B**) SHAP summary plots showing the average impact of each gene on the XGBoost model output, with the x-axis denoting the SHAP value. Positive values push predictions toward one class, whereas negative values push predictions toward the other class. Each point represents a sample, colored according to gene expression (red = high, purple = low). (**C**) SHAP dependence plots showing the correlation of gene expression (x-axis) with its contribution to model prediction (SHAP value, y-axis), with color revealing the expression level of an interacting gene. (**D**) SHAP explanation plot for an individual prediction, showing how each gene’s expression contributes to shifting the model prediction from the base value (E[f(x)]) to the final output probability (f(x)). AUCs were estimated from out-of-fold predictions under cross-validation (internal validation).

To assess the combined diagnostic value of these six PANRGs, nine machine learning models were constructed to distinguish CRC tissues from normal tissues (Figure 6A). The AUCs reported were calculated from cross-validated out-of-fold prediction (internal validation) as opposed to a completely independent external cohort. ROC curve analysis demonstrated strong performance across all the models, with the XGBoost model achieving the highest AUC of 0.981 and being chosen for subsequent interpretation. Accordingly, we have revised the wording to avoid overstating external generalizability based solely on this internal estimate. SHAP was employed for the interpretation of the XGBoost model. The SHAP summary plot (Figure 6B) shows the mean impact of each gene on the output, which indicates that CHP2 was the top feature (mean absolute SHAP value = 0.0356), followed by PIK3CG and CASP7. Each dot represents a sample, and the color represents expression (red: high; purple: low). In the case of CHP2, high expression (red) is usually associated with negative SHAP values, shifting the prediction toward normal tissue, and low expression (purple) with positive SHAP values, shifting the prediction toward tumor tissue. SHAP dependence plots (Figure 6C) show gene interactions. The SHAP value for CHP2 sharply changes around an expression level of ~7.5, which is modified by PIK3CG expression. Finally, the individual-sample prediction plot (Figure 6D) allows us to determine how the model is making predictions: the base value (E[f(x)] = 0.931) is the average prediction value of the dataset. For this sample, low expression of CHP2 (3.67) was the largest positive SHAP value (+0.0201), cumulatively increasing the final prediction probability to 0.998, which resulted in a certain classification.

### 3.6. CHP2 as a Prognostically Related Tumor Suppressor and Functional Pathway Analysis

**Figure 7:**
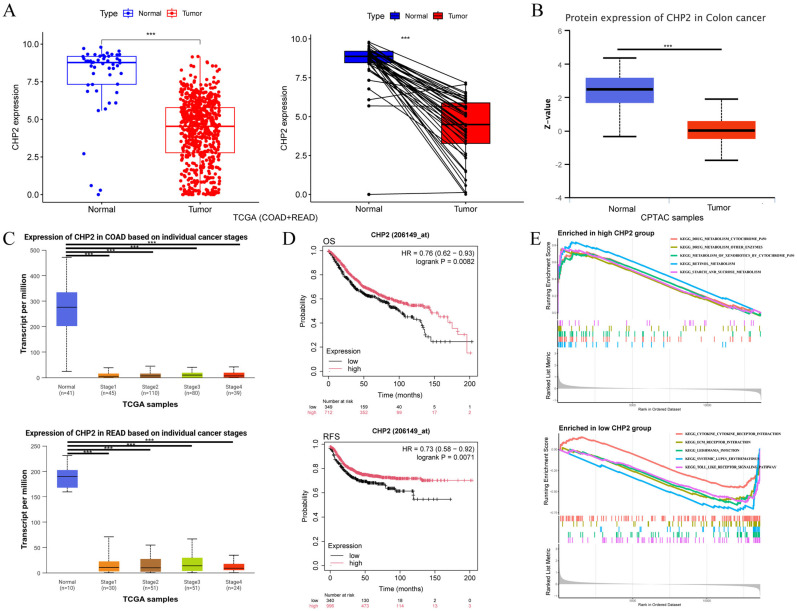
Clinical relevance and functional pathway analysis of CHP2 in CRC. (**A**) CHP2 mRNA levels in normal versus tumor tissues from the TCGA (COAD + READ) cohort. (**B**) CHP2 protein levels in normal and tumor tissues according to the UALCAN database. (**C**) CHP2 expression across clinical stages of CRC from the UALCAN database. (**D**) K-M survival curves showing the associations between CHP2 levels and overall survival and relapse-free survival in CRC patients. (**E**) GSEA-KEGG pathway enrichment analysis comparing the high- and low-CHP2 expression groups. *** *p* < 0.001.

To conduct a systematic investigation of the clinical value of CHP2 in CRC, a group of validation analyses were performed (Figure 7). In the TCGA (COAD + READ) dataset, the mRNA expression of CHP2 was significantly lower in tumor tissues than in normal tissues (Figure 7A), which was verified at the protein level in the CPTAC dataset (Figure 7B). When examined across clinical stages, CHP2 was significantly decreased from normal to Stage I and maintained low expression throughout Stages II, III and IV (Figure 7C). K-M survival analysis revealed that a higher CHP2 level was associated with improved overall survival (*p* = 0.0082) and relapse-free survival (*p* = 0.0071) (Figure 7D). GSEA-KEGG analysis highlighted pathways associated with CHP2 expression. In CHP2-low tumors, immune- and inflammation-related pathways, including KEGG_CYTOKINE_CYTOKINE_RECEPTOR_INTERACTION, KEGG_LEISHMANIA_INFECTION, KEGG_ECM_RECEPTOR_INTERACTION, KEGG_SYSTEMIC_LUPUS_ERYTHATOSUS, and KEGG_TOLL_LIKE_RECEPTOR_SIGNALING_PATHWAY, were enriched. Conversely, CHP2-high tumors were enriched in metabolic pathways, including KEGG_DRUG_METABOLISM_CYTOCHROME_P450, KEGG_DRUG_METABOLISM_OTHER_ENZYMES, KEGG_METABOLISM_OF_XENOBIOTICS_BY_CYTOCHROME_P450, KEGG_RETINOL_METABOLISM, and KEGG_STARCH_AND_SUCROSE_METABOLISM (Figure 7E).

### 3.7. Prediction of Drug Sensitivity Based on CHP2 Expression in CRC

**Figure 8:**
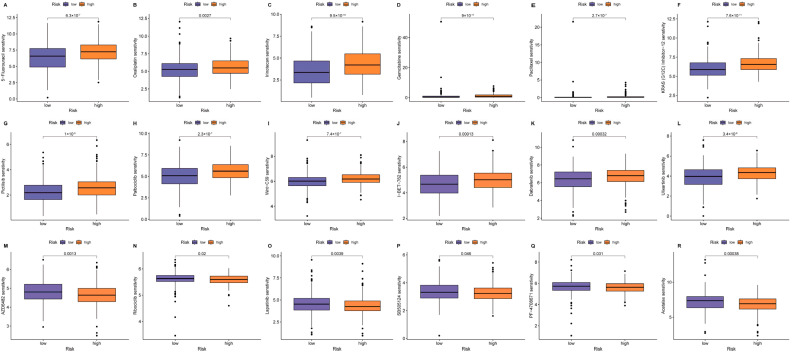
Drug sensitivity prediction based on the risk model. Boxplots illustrating differences in drug sensitivity, measured by the IC_50_, between the high-risk (orange) and low-risk (purple) CRC groups across 18 therapeutic agents. Higher IC_50_ values indicate lower sensitivity (greater resistance). IC_50_ comparisons for the high-risk group are shown for (**A**) 5-fluorouracil, (**B**) oxaliplatin, (**C**) irinotecan, (**D**) gemcitabine, (**E**) paclitaxel, (**F**) KRAS (G12C) inhibitor-12, (**G**) pictilisib, (**H**) palbociclib, (**I**) Wnt-C59, (**J**) I-BET-762, (**K**) dabrafenib, (**L**) ulixertinib, (**M**) AZD6482, (**N**) ribociclib, (**O**) lapatinib, (**P**) SB505124, (**Q**) PF-4708671, and (**R**) acetalax.

To investigate whether our risk model could predict CRC sensitivity to different therapeutic agents, we examined the differences in the IC_50_ values for 18 chemotherapeutic and targeted drugs between the high- and low-risk groups. Higher IC_50_ values indicate lower drug sensitivity (greater resistance). The analysis revealed a complex, drug-dependent “selective sensitivity/resistance” profile (Figure 8). In this study, the low-risk group was defined by high CHP2 expression, whereas the high-risk group was defined by low CHP2 expression. The high-risk groups exhibited significant resistance to 12 agents and hypersensitivity to the other 6 agents. For classic CRC chemotherapeutic drugs, higher IC_50_ values were observed for core drugs in first-line CRC chemotherapy regimens, including 5-fluorouracil (5-FU, *p* = 6.3 × 10^−7^), oxaliplatin (*p* = 0.0027), and irinotecan (*p* = 9.5 × 10^−10^) (Figure 8A–C). Resistance extended to broad-spectrum chemotherapeutics and other targeted drugs, such as gemcitabine (*p* = 9 × 10^−11^) and paclitaxel (*p* = 2.7 × 10^−7^) (Figure 8D,E). Similarly, the high-risk group showed resistance to inhibitors targeting key oncogenic pathways: KRAS inhibitors, ulixertinib, PI3K, CDK4/6, Wnt,; BET, and BRAF (Figure 8F–L). However, strikingly, the other high-risk groups (Figure 8M–R) presented greater drug sensitivity (lower IC_50_) to six targeted drugs, including the PI3K inhibitor AZD6482 (*p* = 0.0013), the CDK4/6 inhibitor ribociclib (*p* = 0.02), the EGFR/HER2 inhibitor lapatinib (*p* = 0.0039), the TGF-β inhibitor SB505124 (*p* = 0.046), the S6K1 inhibitors PF-4708671 (*p* = 0.031) and acetalax (*p* = 0.00038) (Figure 8M–R). In summary, our results suggest that CHP2 expression predicts various drug sensitivity responses, with low CHP2 expression being associated with predicted resistance to standard chemotherapy but sensitivity to certain targeted agents, such as lapatinib and axitinib.

### 3.8. Experimental Validation of CHP2 as a Functional Biomarker in CRC In Vitro and In Vivo

**Figure 9:**
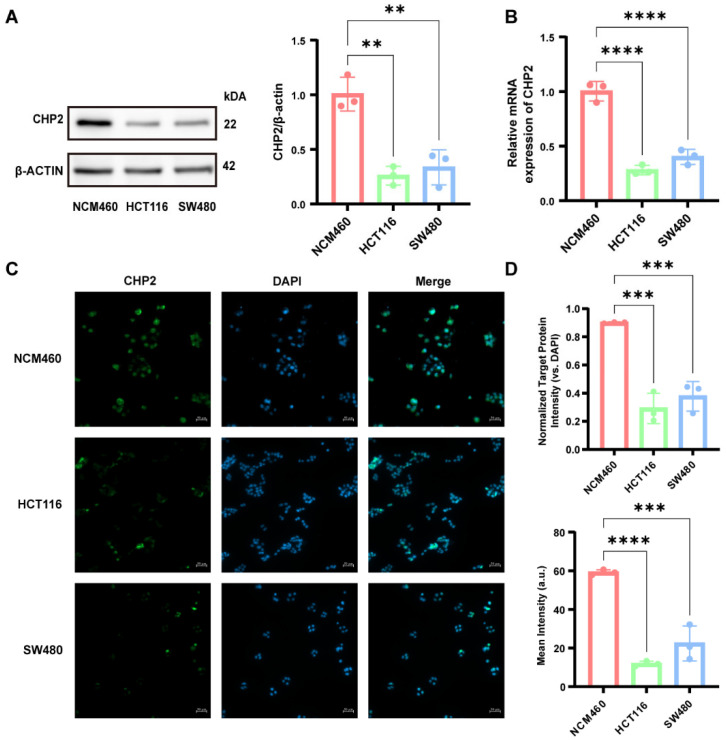
CHP2 downregulation in CRC cell lines. (**A**) Western blot detection and quantification of the protein expression levels of CHP2 in NCM460, HCT116 and SW480 cells. (**B**) The relative mRNA levels of CHP2 in the specified cell lines were measured via qRT-PCR. (**C**) IF staining of CHP2 (green) expression and localization in NCM460, HCT116 and SW480 cells. Nuclei were counterstained with DAPI (blue). (**D**) Quantification of normalized target protein intensity and mean intensity. Statistical significance: ** *p* < 0.01, *** *p* < 0.001, **** *p* < 0.0001.

**Figure 10:**
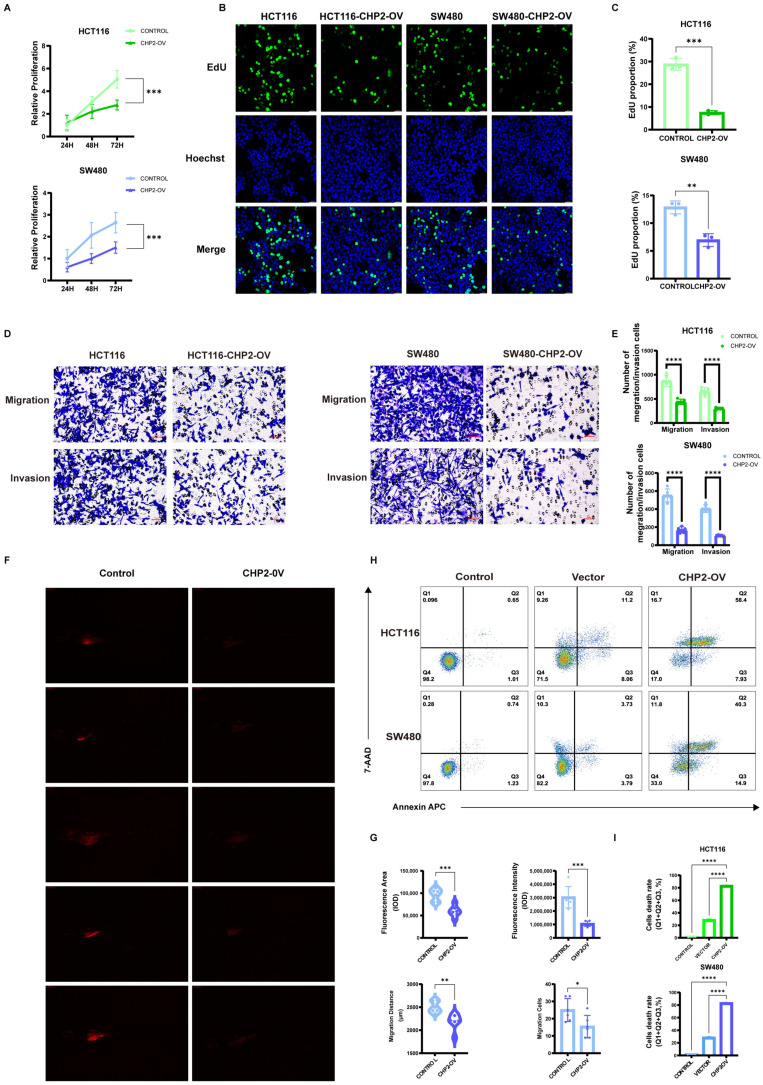
In vitro and in vivo functional validation of the CHP2 gene in CRC. CHP2 overexpression inhibits malignant phenotypes in vitro and in vivo. (**A**) CCK8 assays showing the effect of CHP2 overexpression (CHP2-OV) in HCT116 and SW480 cells. (**B**,**C**) EdU assay (**B**) and the number of HCT116 and SW480 cells (**C**) that underwent proliferation with CHP2-OV. (**D**,**E**) Transwell (**D**) and statistical analyses (**E**) of the migration and invasion of HCT116 and SW480 cells with CHP2-OV cells. (**F**,**G**) Zebrafish xenograft (**F**) and quantification (**G**) of the impact of CHP2-OV on in vivo proliferation and migration. (**H**,**I**) Flow cytometric analysis (**H**) and quantitation of the death rates (Q1 + Q2 + Q3) (**I**) of HCT116 (**top**) and SW480 (**bottom**) cells with CHP2-OV. Statistical significance: * *p* < 0.01, ** *p* < 0.01, *** *p* < 0.001, **** *p* < 0.0001.

To validate the bioinformatics predictions, we assessed CHP2 expression in the normal colon epithelial cell line NCM460 and the CRC cell lines HCT116 and SW480. Western blotting, qRT-PCR, and immunofluorescence consistently demonstrated that both CHP2 protein and mRNA levels were significantly lower in CRC cells than in NCM460 cells (Figure 9A–D). To investigate its biological function, we overexpressed CHP2 in HCT116 and SW480 cells. EdU and CCK-8 assays revealed that CHP2 overexpression significantly inhibited cancer cell proliferation (Figure 10A–C). Transwell assays further demonstrated that CHP2 overexpression markedly attenuated the migratory and invasive capabilities of both HCT116 and SW480 cells (Figure 10D,E). Flow cytometry analysis (Figure 10H,I) via Annexin APC/7-AAD staining revealed that CHP2 overexpression significantly promoted programmed cell death, as evidenced by a significant increase in the single-positive and double-positive cell populations (Q1, Q2, and Q3). Given that there is extensive crosstalk between the pathways of apoptosis, pyroptosis and necroptosis and that CHP2 may be involved in several signaling pathways, we speculate that the overexpression of CHP2 might lead to a complex, mixed form of PCD similar to PANoptosis. Finally, we used a zebrafish xenograft model to confirm these results in vivo (Figure 10F,G). These results indicate that the proliferation and migration capacities of CHP2-OV cells injected into zebrafish were significantly impaired compared with those of control cells. These observations were recapitulated in the fluorescent area and intensity of the tumor and in the migration distance and number of tumor cells inside the zebrafish.

### 3.9. Validation of CHP2 as a Predictive Biomarker for Drug Sensitivity and Its Potential to Induce PANoptosis

**Figure 11:**
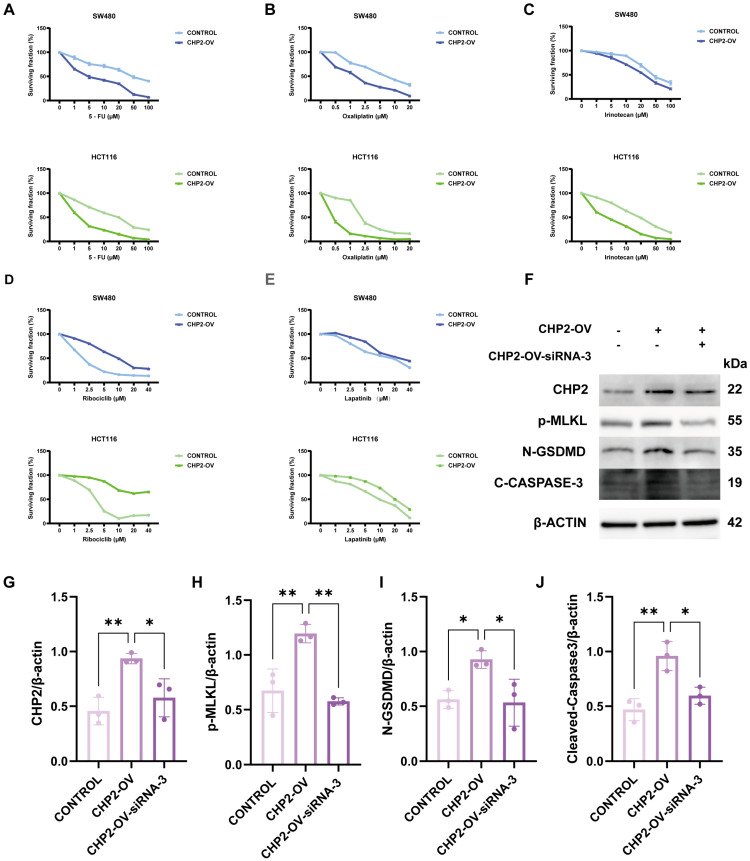
Experimental validation of CHP2 as a predictive biomarker for drug sensitivity and a driver of PANoptosis. (**A**–**C**) IC_50_ growth inhibition curves of HCT116 and SW480 cells treated with first-line chemotherapeutic agents: (**A**) 5-fluorouracil (5-FU), (**B**) Oxaliplatin, and (**C**) Irinotecan. (**D**,**E**) IC50 curves for targeted agents: (**D**) Ribociclib and (**E**) Lapatinib. (**F**) Western blot analysis of key PANoptosis markers, including p-MLKL (necroptosis), N-GSDMD (pyroptosis), and Cleaved-Caspase 3 (apoptosis), in Control, CHP2-OV, and CHP2-OV-siRNA-3 groups. (**G**–**J**) Quantitative analysis of protein expression levels normalized to β-actin. Data are presented as mean ±SD from three independent experiments. * *p* < 0.05, ** *p* < 0.01.

In order to assess the predictive value of CHP2, in vitro sensitization assays against both standard and representative targeted agents were conducted. For the standard chemotherapeutic agents 5-fluorouracil (5-FU), oxaliplatin and irinotecan, control cells with low CHP2 expression displayed a resistant phenotype with higher survival fractions. By contrast, overexpression of CHP2 (CHP2-OV) markedly enhanced the sensitivity of HCT116 and SW480 cells to the traditional treatments (Figure 11A–C). On the other hand, a distinct “selective sensitivity” pattern emerged with targeted agents. Low-CHP2 cells were more sensitive to Ribociclib and Lapatinib than the CHP2-OV group (Figure 11D,E). Taken together, these results indicate that levels of CHP2 may be a potential biomarker for differentiating therapeutic responses between traditional chemotherapeutic agents and certain targeted agents.

To further elucidate whether CHP2 regulates the crosstalk between different RCD modes, we performed Western Blot assays to detect the terminal executioners of the three PANoptic pathways: p-MLKL (necroptosis), N-GSDMD (pyroptosis), and Cleaved-Caspase3 (apoptosis). Given that these proteins serve as the definitive molecular endpoints for their respective pathways, their simultaneous detection allows for a robust confirmation of the PANoptotic process rather than an isolated cell death event. Western blotting analysis revealed that CHP2-OV simultaneously upregulated the levels of PANoptosis markers p-MLKL (necroptosis), N-GSDMD (pyroptosis), and Cleaved-Caspase3 (apoptosis) (Figure 11F–J). Rescue experiments, in which this effect was partially rescued by CHP2-specific siRNA, implying that CHP2 might serve as a regulator in the PANoptosis network. Three independent replicates were performed for each marker, and the original uncropped blots are presented in Figure S6.

## 4. Discussion

The heterogeneity of CRC is a major cause of diverse responses to therapy and drug resistance. Cancer cells can survive by escaping PCD, which we have learned is inherently related to the tumor’s immunological condition. This results in “hot” vs. “cold” tumors. Nevertheless, the molecular switches that regulate this phenotype and how they are related to cell death are poorly understood. We appreciate that conventional investigations have been piecemeal, too often isolating apoptosis to avoid the many interconnects that exist in the modalities of cell death. We propose that PANoptosis, which is a regulated assembly that integrates apoptosis, pyroptosis, and necroptosis, provides a more comprehensive model. We anticipate that this network might connect a tumor’s intrinsic cell death competence to its extrinsic immune context. Here, by simultaneously investigating related collective dysregulation, we intend to identify master regulators that determine whether a tumor becomes adjuvant resistant and “cold”. This PANoptosis-oriented strategy brought us to CHP2, an entirely unknown gene in CRC. Our data conclusively demonstrate that the downregulation of CHP2 is a central event associated with poor outcomes, the establishment of an immunosuppressive “cold” microenvironment, and a complex pattern of selective drug responsiveness. This work substantiates the PANoptosis paradigm in CRC and establishes CHP2 as a determinant of the immunologic and therapeutic fate of tumors.

Our work highlights the potential significance of CHP2 in associating with the CRC TIME. The TIME is a multifaceted ecosystem. The balance between pro- and anti-tumor immune cells determines treatment responses [62]. Our integrative analysis suggests that the downregulation of CHP2 may represent a characteristic feature of an immunosuppressive “cold” tumor state in CRC. This association may be characterized by two potential patterns of immune landscape alteration. First, the diminished expression of CHP2 coincides with a possible shift in the adaptive immune response, evidenced by its positive correlation with plasma cells, naive B cells, and resting CD4+ T cells. [57,60]. The loss of CHP2 expression might therefore track with the depletion of these populations, potentially weakening the synergistic interaction between M1 macrophages and T follicular helper (Tfh) cells [59]. Second, downregulation of CHP2 is associated with an environment conducive to pro-tumoral chronic inflammation. This is suggested by its negative correlation with certain innate immune subsets, such as M0 macrophages and resting dendritic cells. An accumulation of these subsets may correspond to a microenvironment characterized by T-cell suppression and enhanced metastatic potential. [57,58,61]. This cellular change driven by molecules is rather remarkable. We found that low CHP2 expression correlated with increased expression of immune checkpoints (e.g., PD-L1 and B7-H3) [63,64,65] and a chemokine signature potentially promoting pro-angiogenic factors such as CXCL8. [66]. the noted associations also indicate that key costimulatory molecules, such as the BAFF/BCMA (TNFRSF13B/TNFRSF17) pathway, might be perturbed in tumors exhibiting reduced CHP2 levels [67]. Together, all these complex observations suggests that the diminished expression of CHP2 is associated with suppressed of antitumor immunity which leads to an unresponsive “cold” tumor. Rather than providing a definitive mechanistic proof, these findings provide a conceptual framework for understanding the association between its expression levels and the specialized immunological remodeling of the colorectal cancer microenvironment.

This research redefines the role of CHP2 as a systemic, multidomain modulator and biomarker in CRC. It was discovered through a systematic PANRG investigation. An ensemble of nine machine learning algorithms was used in this screening process. It had the greatest diagnostic value among all the candidates (AUC = 0.938). We subsequently validated it as a significantly downregulated tumor suppressor in both the TCGA and CPTAC cohorts. Our investigation demonstrated that its loss is an early event, even at Stage I. This loss was significantly associated with advanced clinical stage and independently predicted worse overall and relapse-free survival. Our in vitro and in vivo studies provided mechanistic validation for these observations. We also demonstrated that CHP2 was expressed at dramatically lower levels in CRC cell lines than in normal colon epithelial cells. More importantly, restoration of CHP2 expression dramatically inhibited CRC cell malignant phenotypes, including cell proliferation, migration and invasion. In addition, it strongly induces multiple forms of programmed cell death. This finding provides the first experimental validation of the role of the TIME as a key effector in the PANoptosis network, showing a direct association between its expression levels and cancer cell fate regulation. As previously described, CHP2 also regulates the TIME as a master regulator. Its deficiency reduces a “cold” tumor phenotype. These findings suggest that CHP2 restoration might be a means to render these “cold” tumors “hot” and enhance immunotherapeutic efficacy. In our GSVA and GSEA analyses, we further observed bipolarity in the function of CHP2 in treatment resistance. The downregulation of CHP2 leads to the activation of a number of protumoral pathways, such as “Pathways in Cancer”, “ECM-receptor interaction” and “TLR signaling”. High-level CHP2 expression, however, primes cells for a binary fate. This destiny is associated with both the potential to induce PANoptosis (through nitrogen metabolism [25]) and escape metabolism (through drug metabolism and pH balance). This finding provides a direct molecular rationale for the general chemoresistance we observed. For upstream investigation of CHP2, we established a candidate multimodal ceRNA network based on bioinformatic prediction. suggests a potential interaction network involving LINC00689, hsa-miR-146a-3p, and hsa-miR-1236-3p. In addition, the result suggested a possible connection to the lncRNA FAM74A7, which could be involved in upstream regulation of CHP2. We then discovered that a risk model defined by low CHP2 expression predicts a “selective resistance” profile. This “high-risk (low expression of CHP2)” phenotype is associated with resistance to a broad arsenal of standard therapies, including several first-line chemotherapy regimens for CRC. This observation suggests that CHP2 loss may promote a state of metabolic adaptation or cellular quiescence, which would provide intrinsic resistance to cytotoxic drugs acting on pace of cell division [68]. Paradoxically, the same state confers a “synthetic lethal” sensitivity to a certain class of targeted agents, including PI3Kβ (AZD6482) and CDK4/6 (ribociclib) inhibitors. These findings indicate that in the absence of CHP2, tumor cells may become hyper-dependent on alternative survival signaling pathways (e.g., PI3K or cell cycle checkpoints) and thus become overly sensitive to directed inhibition of these axes [69,70]. With this observation, CHP2 is not only a prognostic marker but also a predictive marker. It is a “molecular compass” that guides personalized therapeutic approach. By identifying patients who are unlikely to respond to standard chemotherapy, CHP2 expression levels could inform the transition toward alternative regimens, such as synthetic lethality-based targeted therapies.

In conclusion, our work provides a new conceptual model for understanding CRC heterogeneity through the lens of PANoptosis rather than isolated cell death pathways. Our rigorous multiomics analysis led to the identification of CHP2, a largely uncharacterized gene, as a key node in the dysregulation of this network in CRC. We also confirmed that CHP2 is an excellent independent prognostic indicator and that reduced expression of CHP2 is an important molecular event in the development of CRC. More importantly, we answer an essential question by discovering the opposite role of CHP2’s expression. Its loss is associated with not only the development of an immunosuppressive “cold” TIME but also a complicated “selective resistance” pattern. This signature confers widespread chemoresistance but interestingly also reveals synthetic lethal susceptibilities to several focused drug classes in the low-CHP2 high risk group. Consistent with our bioinformatic predictions, our in vitro assays confirmed that CRC cells with low CHP2 expression exhibit increased resistance to first-line chemotherapies (such as 5-FU) while showing potential sensitivity to targeted inhibitors like Ribociclib and Lapatinib. However, several limitations are worth mentioning. While we have provided preliminary experimental support, the results were based partially on bioinformatic analysis of public databases. The detailed molecular mechanisms that govern the CHP2 regulatory network (e.g., the predicted ceRNA network) remain to be experimentally validated. In addition, although our drug sensitivity validations are intriguing, these predictions still need to be tested in CRC models derived from patients. Importantly, our Western blot analysis revealed that CHP2 overexpression concurrently activated hallmark markers for necroptosis (p-MLKL), pyroptosis (N-GSDMD), and apoptosis (Cleaved-Caspase3), which was further supported by siRNA-mediated rescue experiments. These findings suggest that CHP2 may act as a potential regulator of the PANoptosis network, offering a more specific and comprehensive mechanistic insight than the observation of any single mode of programmed cell death. Subsequent studies should concentrate on corroborating our PANoptosis-based risk model in clinical trials to determine its practical value for predicting treatment response. Notably, our observations of synthetic lethal pairs may lead to new combination strategies specifically for patients with low CHP2, which results in chemotherapy drug resistance. Our study provides a preliminary approach to turning a “high-risk” drug-resistant CRC patient into a “high-reward” candidate for precision medicine. This approach hinges on the identification of CHP2 as a major determinant of disease outcome and its repositioning as a critical modulator of disease prognosis.

## 5. Conclusions

Our study identified CHP2 as a critical, dual-function biomarker in CRC that can integrate and explain its multifaceted biological processes under the PANoptosis framework. Our findings validate the importance of CHP2 downregulation in poor prognosis in CRC patients. This is a mechanistic consequence of its holistic generation of a “cold” immunosuppressive TME. Importantly, the high-CHP2 status also reveals a “selective resistance” therapeutic weakness, thereby reclassifying CHP2 from a pure prognostic marker to a predictive marker. It can indicate panresistance to many standard drugs. However, it also pinpoints medicines that are specific targets for synthetic lethal relationships. Thus, we recommend a PANoptosis-based risk model for the molecular stratification of patients according to CHP2 expression. Such a translation-derived method might transform “high-risk” drug-resistant patients into a “high-reward” population in precision medicine; however, these results should now be validated in patient cohorts and additional mechanistic studies.

## Data Availability

The original contributions presented in this study are included in the article/Supplementary Material. Further inquiries can be directed to the corresponding author.

## Supplementary Materials

The following supporting information can be downloaded at: https://www.mdpi.com/article/10.3390/cells15050430/s1, Figure S1. Verification of CHP2 over-expression stable cell lines by WB assays. Figure S2. The structure of plv -EF1a- CHP2 -3*Flag -CMV -luc- P2A- Puro. Figure S3. The results of the knockdown experiments of siRNAs. Figure S4. EdU Assays repeated 3 times independently. Figure S5. Uncropped blots with three independent replicates. Figure S6. Uncropped blots with three independent replicates of PANoptosis hallmark. Figure S7. Immunofluorescence assays with three independent experiments to validate the expression of CHP2 between three types cells. Figure S8. Transwell migration and invasion assays. Figure S9. Flow cytometry assays. Figure S10. The relative expression of miRNAs and lncRNAs. (A) hsa-miR-146a-3p; (B) hsa-miR-660-5p; (C)hsa-miR-1236-3p; (D) LINC00689; (E) FAM74A7. Table S1. Detailed list of PANoptosis-related genes (PANRGs) associated with pyroptosis, apoptosis, and necroptosis. Table S2. The 22 subtypes of infiltrating immune cells analyzed by the CIBERSORTx algorithm. Table S3. A curated set of immune-related genes used for correlation analysis with key genes. Table S4. Full sequence of the human CHP2 gene and structural features of the overexpression plasmid. Table S5. Specific primer sequences for mRNAs, lncRNAs, and miRNAs used in qRT-PCR validation. Table S6. Full GO enrichment results for the total set of PANoptosis-related genes (PANRGs). Table S7. Full KEGG enrichment results for the total set of PANRGs. Table S8. List of 38 differentially expressed DE-PANRGs identified in CRC. Table S9. Complete GO enrichment results for the 38 identified DE-PANRGs. Table S10. Complete KEGG enrichment results for the 38 identified DE-PANRGs. Table S11. Detailed feature selection results and importance rankings of diagnostic PANRGs based on the LASSO, SVM-RFE, and RF algorithms. Table S12. Predicted candidate miRNAs targeting the key PANRGs based on the intersection of miRanda, miRDB, and TargetScan databases. Table S13. Potential lncRNAs predicted to sponge the candidate miRNAs identified via the SpongeScan database.
