# Supplementary material for: Machine Learning-Driven Multi-Omics Analysis Identifies CHP2 as a Key PANoptosis-Related Dual-Function Biomarker in Colorectal Cancer
Source: Cells. 2026 Feb 28;15(5):430. doi: 10.3390/cells15050430 (PMC12985049; doi:10.3390/cells15050430)
Supplement: Supplementary file 1 [file cells-15-00430-s001.zip › cells-4072192-SUPPLEMENTARY FIGURES.pdf]

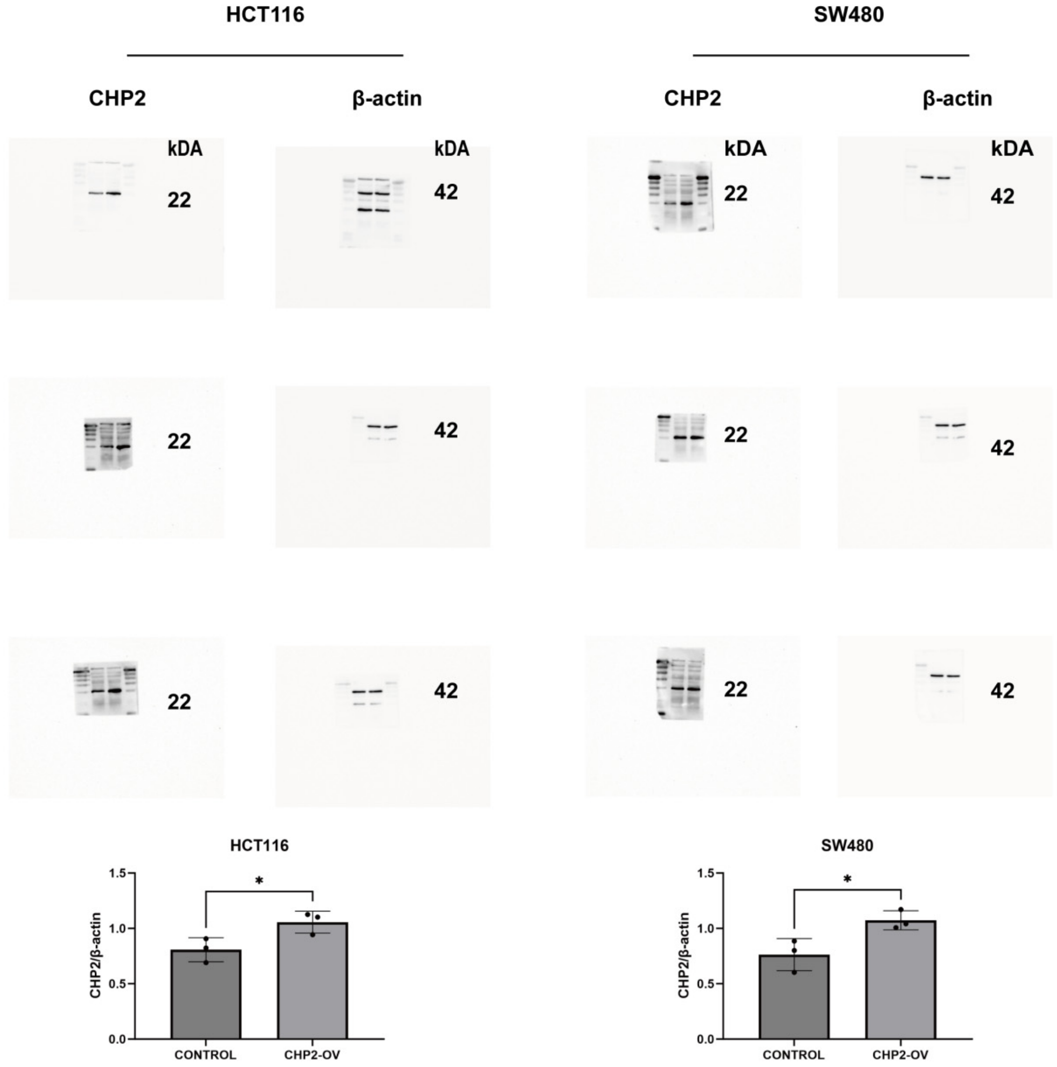

Figure S1. Verification of CHP2 over-expression stable cell lines by WB assays. * *p*<0.05

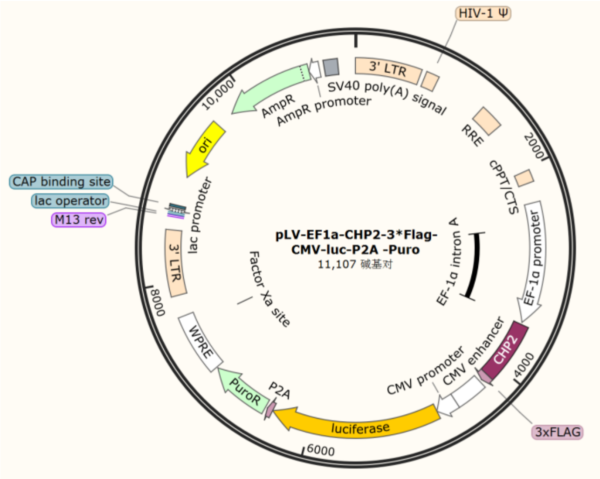

Figure S2. The structure of plv -EF1a- CHP2 -3*Flag -CMV -luc- P2A- Puro.

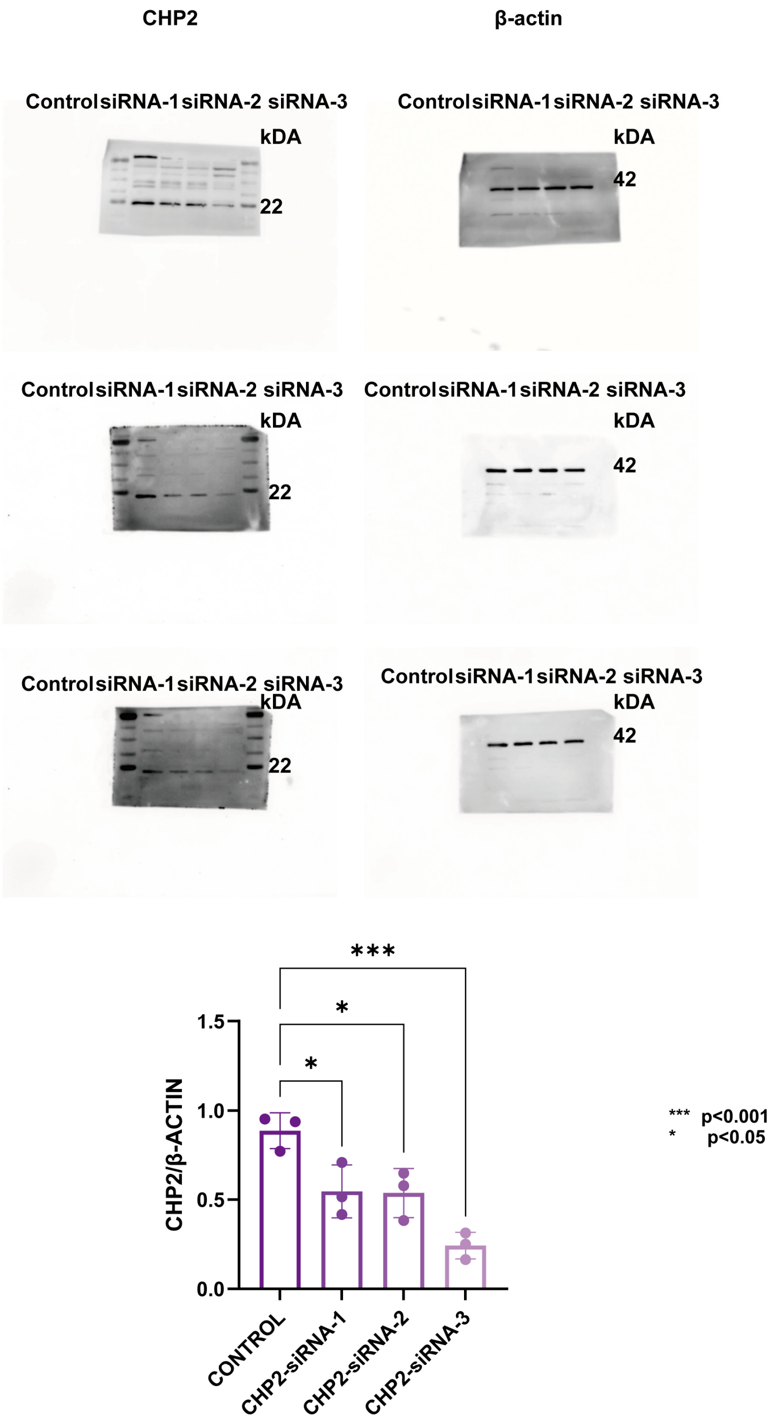

Figure S3. The results of the knockdown experiments of siRNAs.

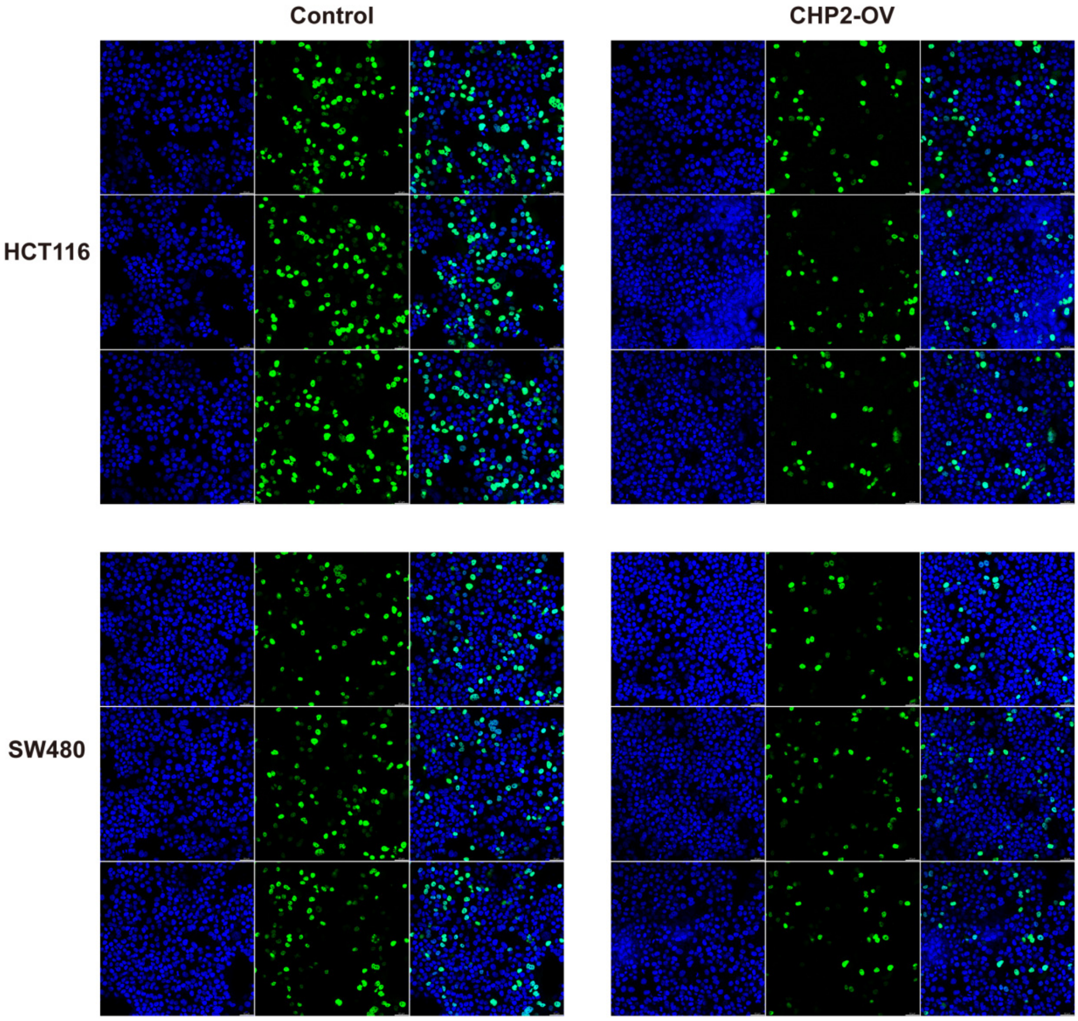

Figure S4. EdU Assays repeated 3 times independently.

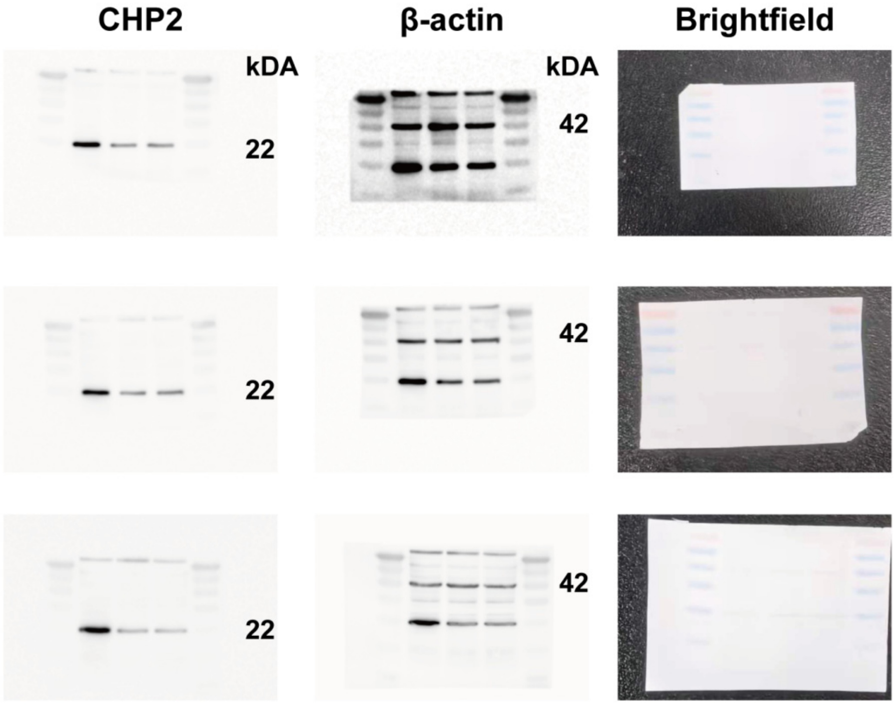

Figure S5. Uncropped blots with three independent replicates.

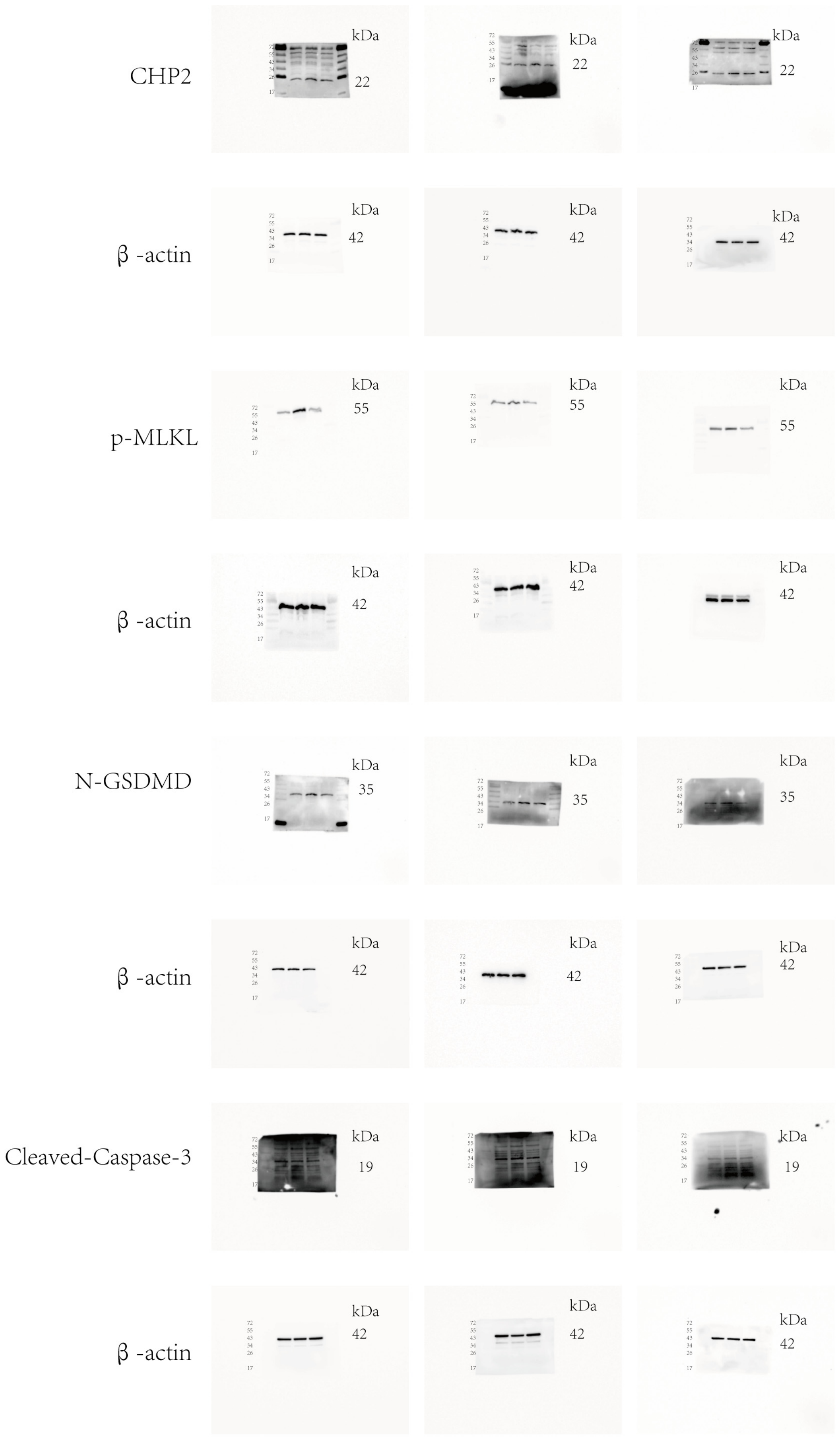
CHP2
kDa
22
β -actin
kDa
42
p-MLKL
kDa
55
β -actin
kDa
42
N-GSDMD
kDa
35
β -actin
kDa
42
Cleaved-Caspase-3
kDa
19
β -actin
kDa
42

Figure S6. Uncropped blots with three independent replicates of PANoptosis hallmark.

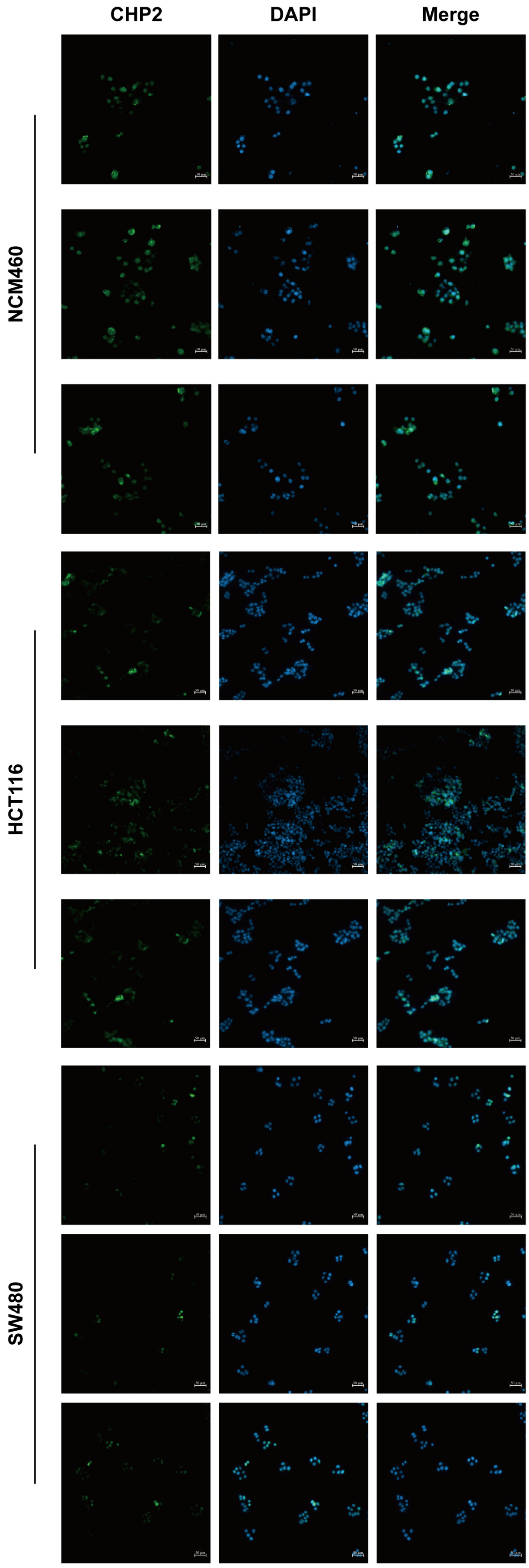
CHP2
DAPI
Merge
NCM460
HCT116
SW480

Figure S7. Immunofluorescence assays with three independent experiments to validate the expression of CHP2 between three types cells.

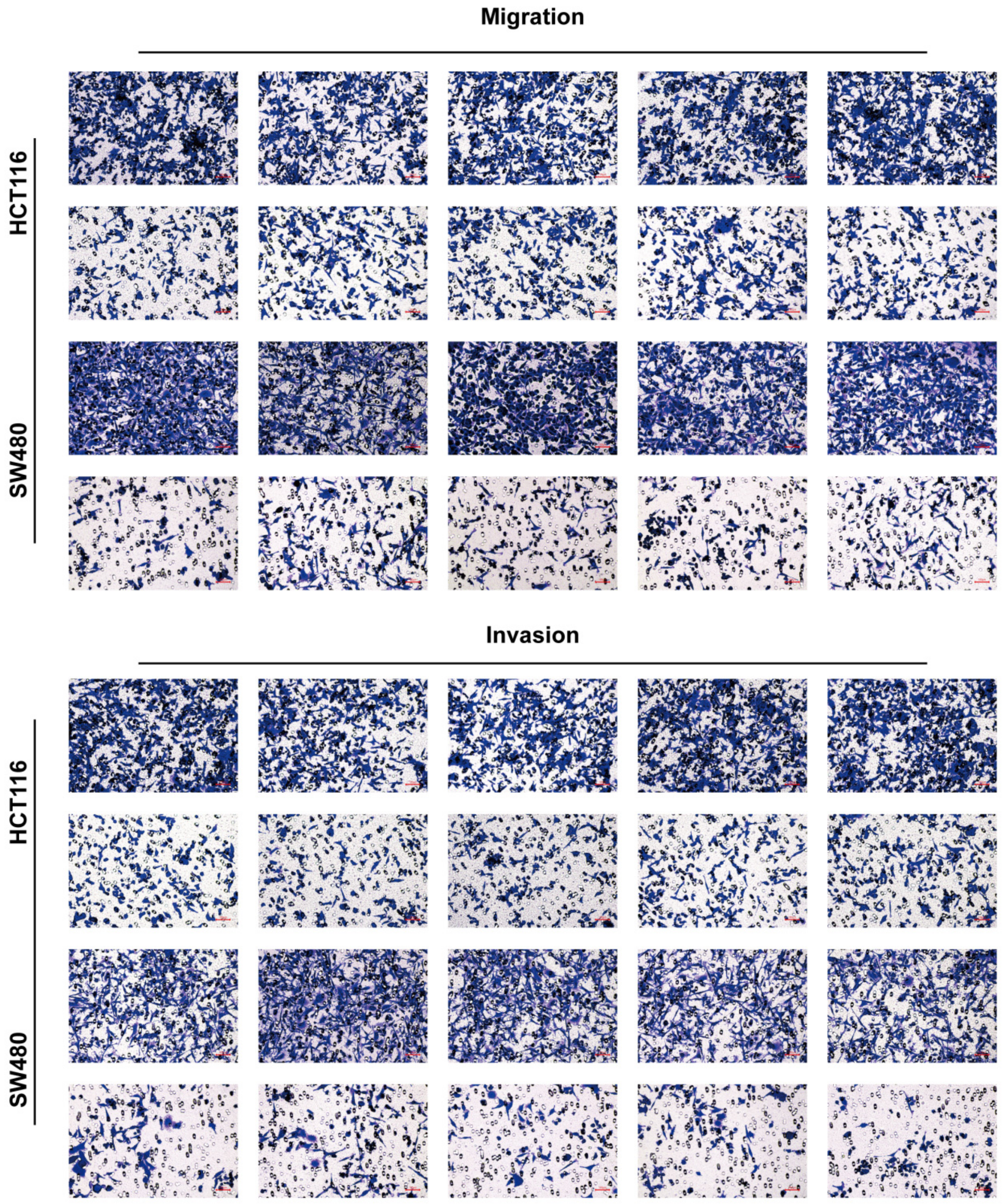

Figure S8. Transwell migration and invasion assays.

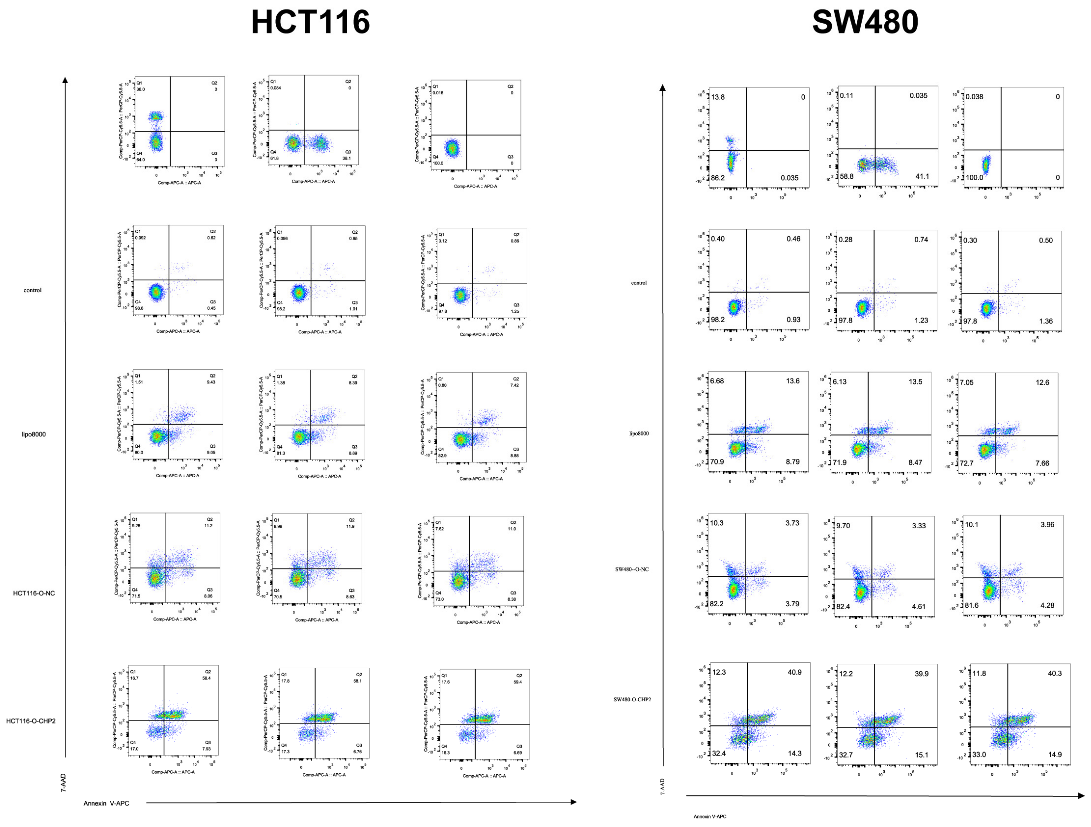

Figure S9. Flow cytometry assays.

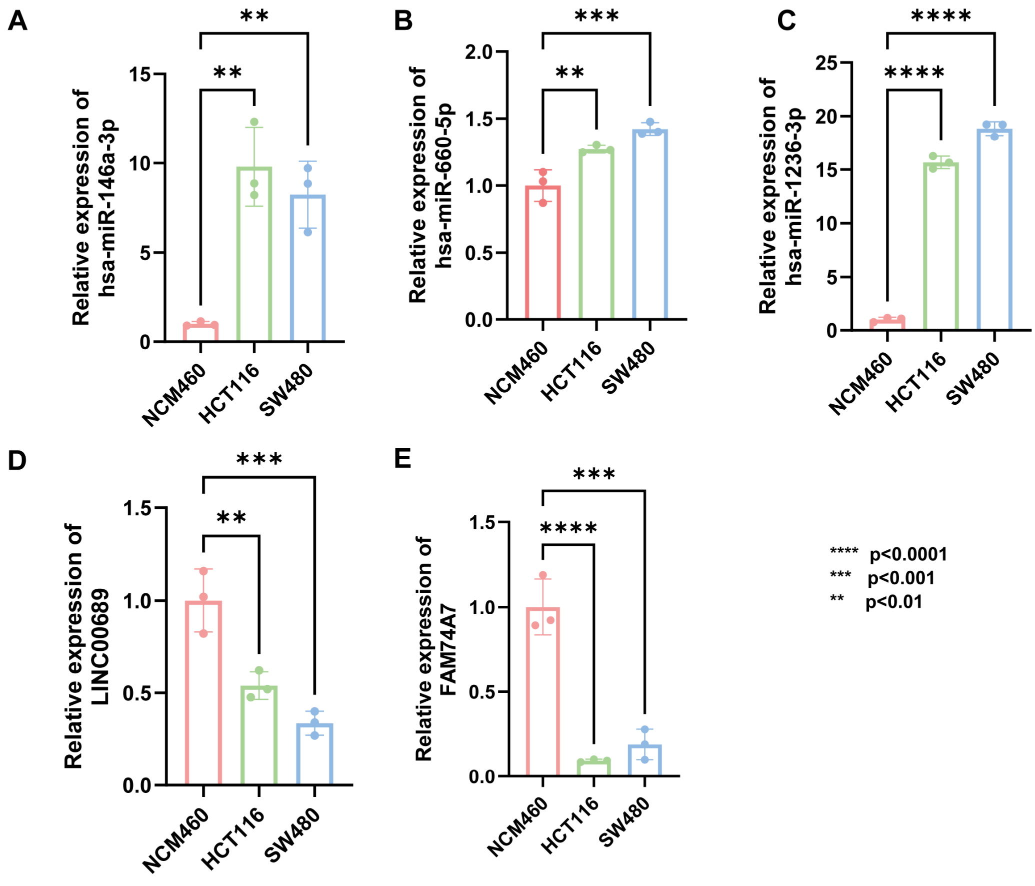

Figure S10. The relative expression of miRNAs and lncRNAs. (A) hsa-miR-146a-3p; (B) hsa-miR-660-5p; (C)hsa-miR-1236-3p; (D) LINC00689; (E) FAM74A7
